# Prevalence and Clinical Implications of a β-Amyloid–Negative, Tau-Positive Cerebrospinal Fluid Biomarker Profile in Alzheimer Disease

**DOI:** 10.1001/jamaneurol.2023.2338

**Published:** 2023-07-31

**Authors:** Pontus Erickson, Joel Simrén, Wagner S. Brum, Gilda E. Ennis, Gwendlyn Kollmorgen, Ivonne Suridjan, Rebecca Langhough, Erin M. Jonaitis, Carol A. Van Hulle, Tobey J. Betthauser, Cynthia M. Carlsson, Sanjay Asthana, Nicholas J. Ashton, Sterling C. Johnson, Leslie M. Shaw, Kaj Blennow, Ulf Andreasson, Barbara B. Bendlin, Henrik Zetterberg

**Affiliations:** 1 Institute of Neuroscience and Physiology, Department of Psychiatry and Neurochemistry, Sahlgrenska Academy, University of Gothenburg, Gothenburg, Sweden; 2 Clinical Neurochemistry Laboratory, Sahlgrenska University Hospital, Gothenburg, Sweden; 3 Graduate Program in Biological Sciences: Biochemistry, Universidade Federal do Rio Grande do Sul, Porto Alegre, Brazil; 4 School of Medicine and Public Health, University of Wisconsin-Madison, Madison; 5 Wisconsin Alzheimer’s Disease Research Center, University of Wisconsin-Madison School of Medicine and Public Health, Madison; 6 Roche Diagnostics GmbH, Penzberg, Germany; 7 Division of Geriatrics and Gerontology, Department of Medicine, University of Wisconsin-Madison School of Medicine and Public Health, Madison; 8 Geriatric Research Education and Clinical Center of the Wm. S. Middleton Memorial Veterans Hospital, Madison, Wisconsin; 9 Institute of Psychiatry, Psychology and Neuroscience, Maurice Wohl Institute Clinical Neuroscience Institute, King’s College London, London, England; 10 NIHR Biomedical Research Centre for Mental Health and Biomedical Research Unit for Dementia at South London and Maudsley NHS Foundation, London, England; 11 Centre for Age-Related Medicine, Stavanger University Hospital, Stavanger, Norway; 12 Department of Pathology and Laboratory Medicine, University of Pennsylvania School of Medicine, Philadelphia; 13 Institute of Neurology, Department of Neurodegenerative Disease, University College London, London, England; 14 UK Dementia Research Institute, University College London, London, England; 15 Hong Kong Center for Neurodegenerative Diseases, Hong Kong, China

## Abstract

**Importance:**

Knowledge is lacking on the prevalence and prognosis of individuals with a β-amyloid–negative, tau-positive (A−T+) cerebrospinal fluid (CSF) biomarker profile.

**Objective:**

To estimate the prevalence of a CSF A−T+ biomarker profile and investigate its clinical implications.

**Design, Setting, and Participants:**

This was a retrospective cohort study of the cross-sectional multicenter University of Gothenburg (UGOT) cohort (November 2019-January 2021), the longitudinal multicenter Alzheimer Disease Neuroimaging Initiative (ADNI) cohort (individuals with mild cognitive impairment [MCI] and no cognitive impairment; September 2005-May 2022), and 2 Wisconsin cohorts, Wisconsin Alzheimer Disease Research Center and Wisconsin Registry for Alzheimer Prevention (WISC; individuals without cognitive impairment; February 2007-November 2020). This was a multicenter study, with data collected from referral centers in clinical routine (UGOT) and research settings (ADNI and WISC). Eligible individuals had 1 lumbar puncture (all cohorts), 2 or more cognitive assessments (ADNI and WISC), and imaging (ADNI only) performed on 2 separate occasions. Data were analyzed on August 2022 to April 2023.

**Exposures:**

Baseline CSF Aβ42/40 and phosphorylated tau (p-tau)181; cognitive tests (ADNI: modified preclinical Alzheimer cognitive composite [mPACC]; WISC: modified 3-test PACC [PACC-3]). Exposures in the ADNI cohort included [^18^F]-florbetapir amyloid positron emission tomography (PET), magnetic resonance imaging (MRI), [^18^F]-fluorodeoxyglucose PET (FDG-PET), and cross-sectional tau-PET (ADNI: [^18^F]-flortaucipir, WISC: [^18^F]-MK6240).

**Main Outcomes and Measures:**

Primary outcomes were the prevalence of CSF AT biomarker profiles and continuous longitudinal global cognitive outcome and imaging biomarker trajectories in A−T+ vs A−T− groups. Secondary outcomes included cross-sectional tau-PET.

**Results:**

A total of 7679 individuals (mean [SD] age, 71.0 [8.4] years; 4101 male [53%]) were included in the UGOT cohort, 970 individuals (mean [SD] age, 73 [7.0] years; 526 male [54%]) were included in the ADNI cohort, and 519 individuals (mean [SD] age, 60 [7.3] years; 346 female [67%]) were included in the WISC cohort. The prevalence of an A−T+ profile in the UGOT cohort was 4.1% (95% CI, 3.7%-4.6%), being less common than the other patterns. Longitudinally, no significant differences in rates of worsening were observed between A−T+ and A−T− profiles for cognition or imaging biomarkers. Cross-sectionally, A−T+ had similar tau-PET uptake to individuals with an A−T− biomarker profile.

**Conclusion and Relevance:**

Results suggest that the CSF A−T+ biomarker profile was found in approximately 5% of lumbar punctures and was not associated with a higher rate of cognitive decline or biomarker signs of disease progression compared with biomarker-negative individuals.

## Key Points

**Question:**

What is the memory clinic prevalence and prognosis of individuals who have a β-amyloid–negative, tau-positive (A−T+) cerebrospinal fluid (CSF) profile?

**Findings:**

In a clinical cohort of 7679 individuals, prevalence of A−T+ CSF was 4.1%. Longitudinally, cognitively unimpaired individuals or individuals with mild cognitive impairment and an A−T+ CSF profile exhibited similar trajectories to individuals with an A−T− CSF profile in relation to cognitive deterioration, brain atrophy, or cerebral glucose metabolism and β-amyloid burden indexed by positron emission tomography.

**Meaning:**

A CSF profile of A−T+ appears to be benign despite being classified as a pathologic change by guidelines; compared with individuals with biomarker-negative CSF, individuals with A−T+ CSF do not have higher rates of cognitive decline or faster disease progression.

## Teaser

This cohort study assesses the memory clinic prevalence and prognosis of individuals with a β-amyloid–negative, tau-positive cerebrospinal fluid biomarker profile.

## Introduction

Cerebrospinal fluid (CSF) analysis is a well-established method to support a clinical diagnosis of Alzheimer disease (AD), with validated and clinically approved biomarkers for β-amyloid (Aβ) pathology, phosphorylated tau (p-tau), and neurodegeneration. In recent research criteria, low Aβ42 or Aβ42/40 ratio and increased concentration of p-tau have been proposed to support the diagnosis of AD,^1^ with the AT(N) system classifying CSF Aβ and p-tau in the “A” and “T” categories, respectively.^2^ A proposed model of AD pathogenesis suggests that biomarkers reflecting Aβ pathology become abnormal before measures of tau pathology, which is now supported by a vast body of evidence.^3^

Normal CSF Aβ in combination with increased p-tau is a finding sometimes seen in clinical settings.^2^ Because p-tau increases are suggested to be AD specific,^4,5^ and increases would not be expected in Aβ-negative individuals, this finding is challenging to interpret. Guidelines vary in the terminology assigned to this finding, mostly encompassing the concept of suspected non-Alzheimer pathology.^6^ For example, studies evaluating the prevalence of CSF biomarker abnormalities suggest that such an A−T+ CSF profile can be found in up to 20% of cognitively unimpaired (CU) older adults.^7,8,9,10^ In addition, available studies show mixed results in the progression of disease-related changes in individuals with this biomarker profile.^7,10,11^ Previously, various propositions have been made to explain its underlying cause, including non-AD tauopathies (ie, primary age-related tauopathy) and altered CSF dynamics, among others.^6,12^ Importantly, these explanations likely vary depending on the biomarker modality being used. Unlike Aβ biomarkers, which seem to provide similar information in terms of “A” status when determined by positron emission tomography (PET) or CSF, tau biomarkers present pronounced differences across imaging and fluid modalities. Although fibrillar tau aggregates targeted by tau-PET radiotracers are likely a more direct proxy of tangle pathology,^13,14^ soluble p-tau measured by immunoassays may reflect a neuronal response to Aβ pathology,^15,16,17,18^ although p-tau is also present in dystrophic neurites, suggesting that it also reflects tau pathology.^19,20^ Although evidence is accumulating that individuals with an A+T− biomarker profile (and to a greater extent, those with both CSF signs of Aβ and soluble tau pathology [A+T+]) are at risk for cognitive decline,^7,10^ less is known about the clinical implications of the CSF A−T+ pattern. Because CSF biomarkers are frequently used in clinical practice, it is important to further understand the implications of patterns not expected within the currently proposed model of AD biomarker progression.

Given the lack of studies directly evaluating the clinical relevance and prevalence of an isolated increase in CSF p-tau, we aimed to assess the prevalence, as well as cognitive and biomarker progression rates, associated with the CSF A−T+ profile, using 4 large data sets.

## Methods

### Design

Four cohorts were used in the present study: University of Gothenburg (UGOT), the Alzheimer Disease Neuroimaging Initiative (ADNI), Wisconsin Registry for Alzheimer Prevention (WRAP), and Wisconsin Alzheimer Disease Research Center (WI-ADRC). For each cohort, a local institutional review board for human research or ethics committee approved of the study. The UGOT cohort consisted of cross-sectional data and was retrospectively compiled using anonymized data from the clinical routine laboratory database at Sahlgrenska University Hospital, Gothenburg, Sweden, based on CSF analyses performed between November 2019 and January 2021. ADNI is a longitudinal multicenter observational study with data being collected between September 2005 and May 2022. WRAP and the Wisconsin Alzheimer Disease Research Center (WI-ADRC) are single-center, longitudinal, observational, cohort studies that collected data between February 2007 and November 2020. The present study followed the Strengthening the Reporting of Observational Studies in Epidemiology (STROBE) reporting guidelines.

### Setting

The UGOT cohort consists of individuals who underwent lumbar puncture primarily in memory and neurology clinics. The ADNI is a study of volunteers with MCI or early AD, as well as CU individuals enrolled at memory clinics.^11^ The WISC sample included longitudinally observed participants enrolled at midlife at the WRAP or the WI-ADRC referral centers.^21^ Due to their similarities in data collection, WRAP and WI-ADRC were combined and analyzed together and will subsequently be denoted as WISC.

### Participants

In the UGOT cohort, all participants were older than 50 years and had data on age, sex, and CSF biomarker concentrations, whereas clinical information on diagnosis and cognitive function was not available. In the well-characterized longitudinal observational cohorts (ADNI and WISC), we included only individuals who were CU or had mild cognitive impairment (MCI; only in ADNI) at the first cognitive evaluation. In the UGOT cohort, no data on participant race and ethnicity were available, as they are not routinely collected by health care services. In the ADNI cohort, race categories included were American Indian or Alaska Native, Asian, Black, Hawaiian, multiracial, and White. In the WISC cohort, individuals self-identifying as White were reported, whereas American Indian or Alaska Native, Asian, Black or African American, or those not identifying as any of the stated races (here indicated as other), were not reported to maintain anonymity for groups with less than 3 individuals. More information on all cohorts used can be found in eMethods 1 in Supplement 1, and detailed inclusion and exclusion criteria have been described for the ADNI^22^ and WISC cohorts.^21^ Written informed consent was obtained from participants in the ADNI and WISC cohorts, and local institutional review boards for human research approved the study. For the UGOT data, in accordance with the Helsinki declaration, an external ethics committee has approved the procedure of using data from this cohort in research purposes.^5^ There were no identifiable data used in this study, and thus, no informed consent was needed.

### CSF Biomarkers

CSF Aβ42/40 ratio and p-tau181 (hereafter referred to as p-tau) were quantified in all participants and were used to define AT status in all 3 cohorts. In the UGOT cohort, CSF concentrations of Aβ42, Aβ40, and p-tau were quantified on the Lumipulse G1200 platform (Fujirebio), a fully automated assay recently approved by the US Food and Drug Administration.^23^ In both the ADNI and WISC cohorts, p-tau was quantified using a fully automated Elecsys assay (Roche), also recently approved by the US Food and Drug Administration. Aβ42 and Aβ40 were measured with mass spectrometry and Elecsys assays in the ADNI and WISC cohorts, respectively. Elecsys Aβ42 is an in vitro diagnostic test approved for use in the European Union and US; Aβ40 was measured using a research use−only Elecsys assay. CSF status determined at baseline lumbar puncture was tested in the WISC analyses. In all cohorts, previously established cohort-specific cutoffs were used. In ADNI, cutoffs of 0.0138 or less and 24 pg/mL or greater were used for Aβ42/40 and p-tau, respectively. In WISC, abnormal biomarker results were determined to be 0.046 or less and 24.8 pg/mL or greater for Aβ42/40 and p-tau, respectively. More information on the CSF collection and quantification of and cutoff determination for Aβ42, Aβ40, and p-tau can be found in eMethods 2 in Supplement 1.

### Imaging Biomarkers

In longitudinal imaging analyses in ADNI participants, we included those who had at least a baseline and a follow-up scan. We evaluated longitudinal trajectories of Aβ [^18^F]-florbetapir, [^18^F]-fluorodeoxyglucose (FDG) PET, and hippocampal volume magnetic resonance imaging (MRI). A brief description of imaging methods can be found in eMethods 3 in Supplement 1.^24,25^ As a secondary analysis, we evaluated summary tau-PET measures between CSF AT groups, to investigate whether elevated p-tau in the absence of Aβ could represent an abnormal tau-PET phenotype. In the WISC sample, a subset of participants underwent [^18^F]-MK6240 tau-PET and were evaluated with an entorhinal cortex region of interest (ROI), as further detailed in eMethods 3 in Supplement 1.^26^ In the ADNI cohort, a subsample of the individuals herein included had available [^18^F]-flortaucipir tau-PET data up to 6 years after the baseline lumbar puncture, and the previously described ADNI meta-temporal ROI was used as the composite region.^27^

### Cognitive Assessments

The cognitive measures used for longitudinal analyses in the 2 observational cohorts were 2 different versions of the preclinical Alzheimer cognitive composite (PACC): the modified PACC (mPACC) in ADNI and a modified 3-test PACC (PACC-3) in WISC.^28,29,30^ The PACC is a sensitive measure for tracking early cognitive decline, composed typically of 3 key domains: episodic memory, executive function, and global cognition.^31,32^ Specific mPACC and PACC-3 details can be found in eMethods 4 in Supplement 1.

### Statistical Analysis

In the UGOT cohort, we estimated the prevalence of the CSF AT profiles across ages. To better visualize the prevalence across different ages, we fitted a locally estimated scatterplot smoothing line (LOESS), which is a flexible nonlinear regression approach. In all cohorts, general prevalence of each CSF AT profile was calculated, and 95% CIs were estimated using 1-sample proportions tests. In the UGOT cohort, we also performed a sensitivity analysis to evaluate if measured concentrations changed over time or if cutoff-related differences influenced the prevalence (eMethods 5 in Supplement 1).

We used linear mixed-effects models to investigate whether CSF AT group modified the longitudinal rate of change in cognitive function in ADNI (mPACC) and WISC (PACC-3), as well as in Aβ-PET, FDG-PET, and MRI-derived hippocampal volume (ADNI only). The models were fitted with random slopes and intercepts on the participant level and included CSF AT group (A−T− as reference), age, sex, *APOE *ε4 carrier status, and years of education, and an interaction term between CSF AT group and time. In the cognition models, a continuous term adjusting for practice effects (ie, number of occasions the outcome tests were taken before last cognitive assessment) was included in both the ADNI and WISC cohorts, with the time being modeled as age. The WISC model also adjusted for cohort membership. In the ADNI cohort, for each outcome, we fitted separate models for CU individuals at baseline and MCI at baseline. To investigate if a more stringent p-tau cutoff influenced the results of the linear mixed-effects models in the ADNI and WISC cohorts, we performed a sensitivity analysis increasing the predetermined p-tau cutoff values by 15%. As a secondary analysis, we cross-sectionally examined differences in tau-PET uptake across CSF AT groups, in the ADNI and WISC cohorts, with linear models, controlling for age, sex, *APOE *ε4 status, and years of education. Pairwise comparisons using Šidák correction to control for family-wise error rate were performed following a significant omnibus test result. *P* values were 2-sided, and the cutoff for significance was set to *P* < .05. Statistical analyses were performed using R software, version 4.1.2 (R Project for Statistical Computing). Data were analyzed on April 2022 to April 2023.

## Results

### Baseline CSF Characteristics

A total of 7679 individuals (mean [SD] age, 71.0 [8.4] years; 4101 male [53%]; 3578 female [47%]) were included in the UGOT cohort, 970 individuals (mean [SD] age, 73 [7.0] years; 526 male [54%]; 444 female [46%]) were included in the ADNI cohort, and 519 individuals (mean [SD] age, 60 [7.3] years; 346 female [67%]; 173 male [33%]) were included in the WISC cohort. The ADNI cohort included 364 CU individuals and 606 individuals with MCI. The WISC cohort included 513 CU individuals and 6 individuals with MCI. In the ADNI cohort, study participants identified with the following race and ethnicity categories: 1 American Indian or Alaska Native (0.3%), 9 Asian (2.3%), 25 Black (6.5%), 1 Hawaiian (0.3%), 7 multiracial (1.8%), and 341 White (88%). In the WISC cohort, 493 individuals (95%) self-identifying as White were reported; individuals identifying as American Indian or Alaska Native, Asian, Black or African American, or other were not reported in order to maintain anonymity for groups with 3 or fewer individuals. Key information on demographic and other variables is displayed in the Table.

**Table.** Baseline Demographics and Clinical Characteristics

Cohort	Biomarker profile				
	All	A−T−	A+T−	A+T+	A−T+
**UGOT**					
Participants, No. (%); 95% CI, %^a^	7679	3241 (42); 41 to 43	1324 (17); 16 to 18	2798 (36); 35 to 38	316 (4.1); 3.7 to 4.6
Age, mean (SD), y	71 (8.4)	69 (9)	73 (7.6)	73 (7.5)	73 (8.7)
Sex, No. (%)					
Female	3578 (47)	1371 (42)	597 (45)	1487 (53)	123 (39)
Male	4101 (53)	1870 (58)	727 (55)	1311 (47)	193 (61)
CSF Aβ42/40, mean (SD)	0.7 (0.62)	0.93 (0.12)	0.57 (0.093)	0.44 (0.094)	1.1 (2.8)
CSF Aβ42, mean (SD), ng/L	660 (353)	803 (340)	438 (175)	515 (193)	1410 (457)
CSF Aβ40, mean (SD), ng/L	9820 (3920)	8620 (3380)	7680 (2720)	11 700 (3650)	15 000 (4230)
CSF p-tau181, mean (SD), ng/L	56 (44)	28 (9.1)	36 (8.6)	97 (48)	66 (19)
**ADNI**					
Participants, No. (%); 95% CI, %^a^	970	386 (40); 37 to 43	165 (17); 15 to 20	338 (35); 32 to 38	81 (8.4); 6.7 to 10
Age at baseline, mean (SD), y	73 (7.0)	71 (6.9)	74 (6.6)	74 (7.0)	73 (7.3)
Sex (female), No. (%)					
Female	444 (46)	178 (46)	68 (41)	159 (47)	39 (48)
Male	526 (54)	208 (54)	97 (59)	179 (53)	42 (52)
Race, No. (%)^b^					
American Indian/Alaska Native	1 (0.3)	0	0	0	1 (0.1)
Asian	9 (2.3)	1 (0.6)	2 (0.6)	0	12 (1.2)
Black	25 (6.5)	4 (2.4)	8 (2.4)	1 (1.2)	38 (3.9)
Hawaiian	1 (0.3)	0	1 (0.3)	0	2 (0.2)
Multiracial	7 (1.8)	4 (2.4)	1 (0.3)	0	12 (1.2)
White	341 (88)	156 (95)	326 (96)	80 (99)	903 (93)
MCI, No. (%)	606 (63)	199 (52)	105 (64)	268 (79)	34 (42)
*APOE *ε4 positive, No. (%)^c^	403 (42)	76 (20)	87 (53)	225 (67)	15 (19)
Education, mean (SD), y	16.2 (2.7)	16.4 (2.7)	15.9 (2.8)	16.0 (2.7)	16.5 (2.7)
CSF Aβ42/40, mean (SD)	0.14 (0.057)	0.20 (0.029)	0.11 (0.023)	0.088 (0.021)	0.19 (0.034)
CSF Aβ42, mean (SD), ng/L	1180 (620)	1500 (509)	725 (273)	794 (280)	2190 (581)
CSF Aβ40, mean (SD), ng/L	8310 (2460)	7640 (2110)	6820 (1840)	9080 (2270)	11300 (2270)
CSF p-tau181, mean (SD), ng/L	256 (13)	16 (3.9)	19 (3.3)	39 (13)	29 (4.8)
mPACC, mean (SD)	−3.5 (4.3)	−1.7 (3.4)	−3.7 (4.2)	−5.9 (4.3)	−1.9 (3.5)
**WISC** ^d^					
Participants, No. (%); 95% CI, %^a^	519	412 (79); 76 to 82	49 (9.4); 7.2 to 12	37 (7.1); 5.2 to 9.7	21 (4.0); 2.6 to 6.1
WRAP, No. (%)	233 (45)	178 (34)	27 (5.2)	17 (3.3)	11 (2.1)
WI-ADRC, No. (%)	286 (55)	234 (45)	22 (4.2)	20 (3.8)	10 (1.9)
Age, mean (SD), y	60 (7.3)	59 (6.9)	63 (6.2)	66 (7.8)	65 (8.6)
Age at baseline LP, y	62 (7.5)	61 (7.1)	65 (5.9)	69 (7.2)	68 (8.6)
Sex, No. (%)					
Female	346 (67)	275 (67)	31 (63)	23 (62)	17 (81)
Male	173 (33)	137 (33)	18 (37)	14 (38)	4 (19)
Race, No. (%)^b^					
American Indian/Alaska Native	NR	NR	NR	NR	NR
Asian	NR	NR	NR	NR	NR
Black/African American	NR	NR	NR	NR	NR
White	493 (95)	390 (95)	47 (96)	35 (95)	21 (100)
Other	NR	NR	NR	NR	NR
MCI at baseline LP, No. (%)	6 (1.2)	3 (0.7)	0 (0.0)	1 (2.7)	2 (9.5)
*APOE *ε4 positive, No. (%)^c^	194 (37)	138 (34)	30 (61)	23 (62)	3 (14)
Education, mean (SD), y	16.2 (2.4)	16.2 (2.5)	15.8 (2.4)	16.6 (2.2)	16.4 (1.9)
CSF Aβ42/40, mean (SD)	0.06 (.02)	0.07 (.01)	0.04 (.01)	0.03 (.01)	0.06 (.01)
CSF Aβ42, mean (SD), ng/L	927 (379)	977 (347)	495 (148)	608 (226)	1513 (195)
CSF Aβ40, mean (SD), ng/L	14 421 (4714)	13 786 (4241)	12 947 (3474)	18 128 (3977)	23 794 (4146)
CSF p-tau181, mean (SD), ng/L	18 (6.9)	15 (4.3)	19 (3.9)	32 (7.4)	31 (5.6)
PACC-3, mean (SD)	0.09 (.94)	0.13 (.93)	−0.02 (1.02)	−0.12 (.89)	−0.01 (.93)

Abbreviations: Aβ42/40, β-amyloid 1-42 to β-amyloid 1-40; CSF, cerebrospinal fluid; LP, lumbar puncture; MCI, mild cognitive impairment; mPACC, modified preclinical Alzheimer cognitive composite; NR, not reported; p-tau, phosphorylated tau; PACC-3, modified 3-test preclinical Alzheimer cognitive composite.

^a^ 95% CIs were estimated using a 1-sample proportions test.

^b^ Participants who did not self-identify as White identified as American Indian or Alaska Native, Asian, Black or African American, or other. Numbers are NR to maintain anonymity for groups with fewer than 3 individuals.

^c^ Indicates the proportion of individuals carrying at least 1 copy of the *APOE* ε4 allele.

^d^ In the WISC cohort, demographic and clinical characteristics, except where otherwise noted, are from time point of first cognitive evaluation. CSF data are from the time point of first LP.

### Prevalence of CSF AT Profiles in a Real-World Clinical Routine Setting

In the UGOT cohort, 316 of 7679 individuals (4.1%; 95% CI, 3.7%-4.6%) displayed an A−T+ profile, whereas the prevalence of A−T− was 3241 of 7679 (42%; 95% CI, 41%-43%), the prevalence of A+T− was 1324 of 7679 (17%; 95% CI, 16%-18%), and the prevalence of A+T+ was 2798 of 7679 (36%; 95% CI, 35%-38%). Similar estimates were found in the ADNI and WISC cohorts (Table). Alternatively, if determining “A” by CSF Aβ42 alone, as done in several studies discussed subsequently, the prevalence of an A−T+ profile would be 19% (95% CI, 18%-20%) and 37% (95% CI, 36%-38%), 22% (95% CI, 21%-23%), and 21% (95% CI, 20%-22%) for A−T−, A+T−, and A+T+ profiles, respectively.

**Figure 1.**
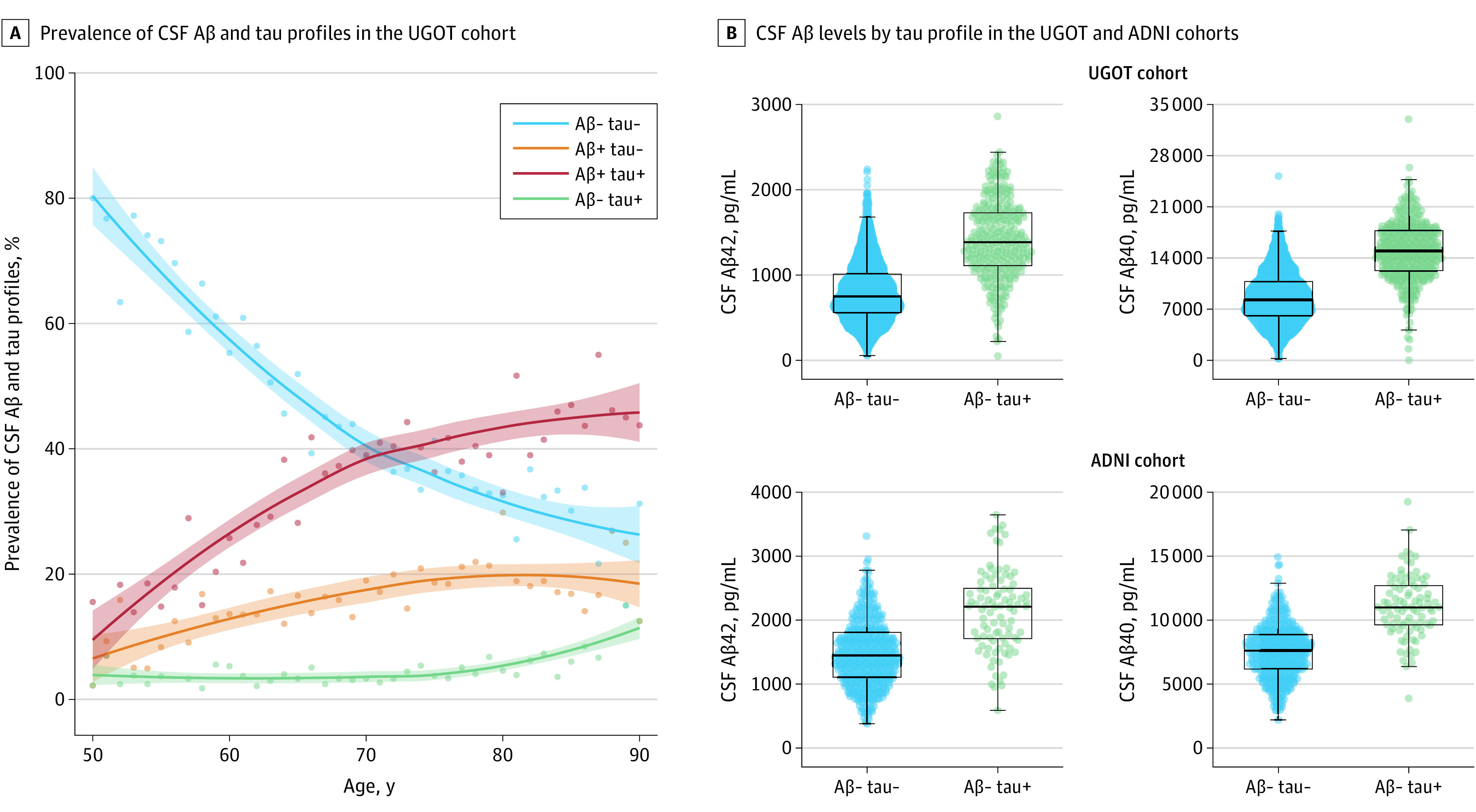
Prevalence Estimates in a Clinical Laboratory Routine Setting Across Ages and Cerebrospinal Fluid (CSF) β-Amyloid 42 (Aβ42) and Aβ40 Concentrations Among Amyloid-Negative Individuals A, The color dots represent the prevalence in percentage at each age (in years) of each biomarker category based on the cutoffs used in clinical routine. The solid lines represent corresponding locally estimated scatterplot smoothing (LOESS) regression lines, with shaded areas indicating 95% CIs. B, The graphs display group comparisons Aβ42 and Aβ40 for the Aβ-negative amyloid-tau (AT) biomarker profiles in both the University of Gothenburg (UGOT) and Alzheimer’s Disease Neuroimaging Initiative (ADNI) cohorts. Group comparisons were performed with linear regression models adjusting for relevant available covariates (UGOT: age, sex; ADNI: age, sex, *APOE* ε4 status, years of education). In the UGOT cohort, mean levels of CSF Aβ42 were significantly increased in individuals with an A−T+ profile by a mean of 627 pg/mL (95% CI, 586-667 pg/mL; *P* <.001), and CSF levels of Aβ40 were significantly increased in the A−T+ group by a mean of 6504 pg/mL (95% CI, 6104-6904 pg/mL; *P* <.001). In ADNI, mean levels of CSF Aβ42 were significantly increased in individuals with an A−T+ profile by a mean of 673 pg/mL (95% CI, 554-793 pg/mL; *P* <.001), and CSF levels of Aβ40 were significantly increased in the A−T+ group by a mean of 3543 pg/mL (95% CI, 3054-4054 pg/mL; *P* <.001).

In visual interpretation of age-stratified LOESS curves, we found that although the prevalence of other CSF profiles changed dynamically toward abnormality with aging, whereas the prevalence of the A−T+ profile was stable with older age, with a minor increase in the age prevalence after 75 years (Figure 1A). In contrast, when performing the same analysis with Aβ42 alone as “A,” a different trend was observed. The age-stratified rates of an A−T+ profile displayed a similar trend to that of A+T− and A+T+ profiles, with all presenting increased prevalence with increased age, reaching similar figures of approximately 25% each in the oldest individuals (eFigure 1 in Supplement 1). In a grayscale analysis to evaluate potential analytical effects with more stringent cutoff values and variation in analytical performance over time, no such significant changes were observed, and very minor changes were found in the prevalence of the A−T+ group (eTable 1 and eFigure 2 in Supplement 1). Interestingly, in both the UGOT and ADNI cohorts, concentrations of CSF Aβ42 and Aβ40 alone were substantially higher in the A−T+ group compared with A−T− (Figure 1B-E).

### Cognitive Trajectories

**Figure 2.**
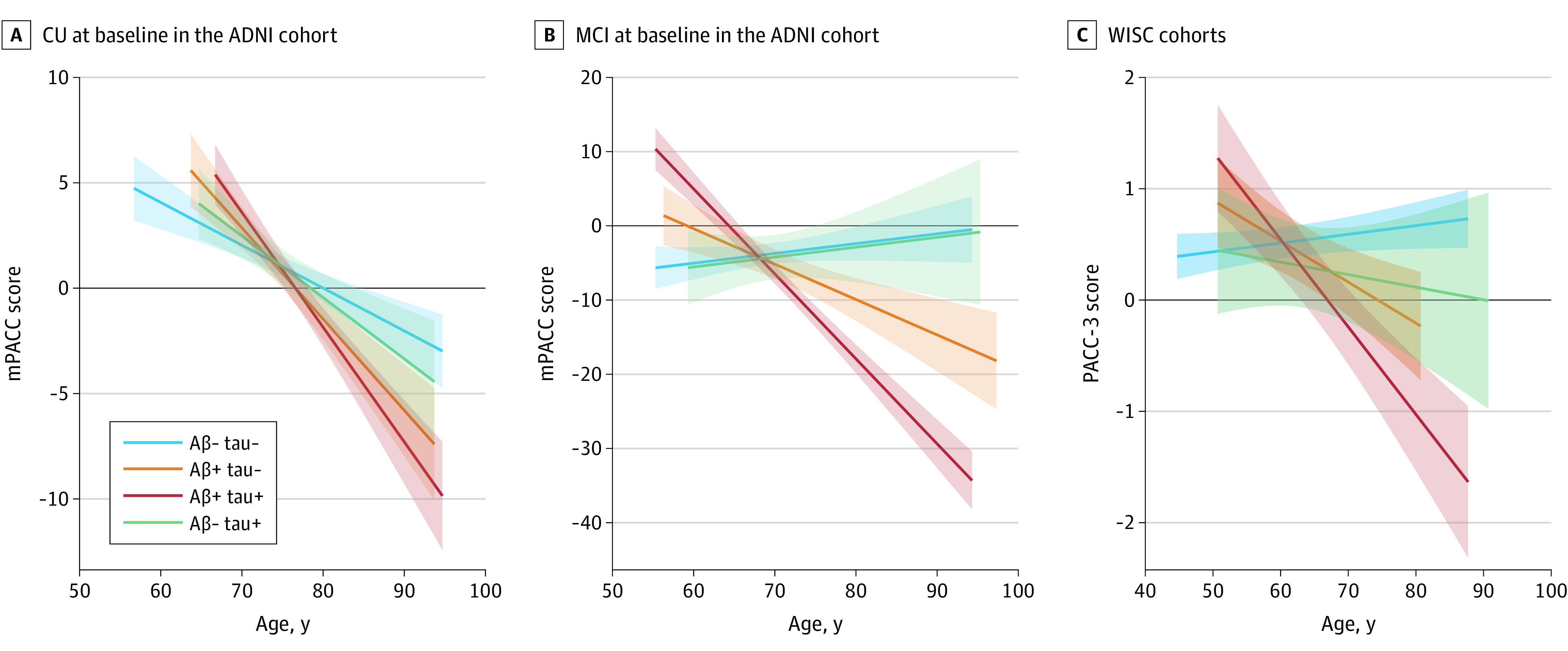
Associations Between Cerebrospinal Fluid (CSF) Amyloid-Tau (AT) Status and Longitudinal Cognitive Decline Mean predicted trajectories of cognitive decline according to CSF AT status and baseline cognitive status are shown for individuals who were cognitively unimpaired (CU) (A) or had mild cognitive impairment (MCI) (B) at baseline in the Alzheimer’s Disease Neuroimaging Initiative (ADNI) and in 2 Wisconsin cohorts, Wisconsin Alzheimer Disease Research Center and Wisconsin Registry for Alzheimer Prevention (WISC) (C). The mean predicted trajectories for the modified Preclinical Alzheimer’s Cognitive Composite (mPACC; ADNI) and modified 3-test PACC (PACC-3; WISC) are displayed with solid lines and 95% CIs. Trajectories were estimated including terms for CSF AT status, covariates (age, years of education, *APOE* ε4 genotype, sex, and practice [and cohort in WISC]), as well as the age × CSF AT status interaction. A+/− indicates CSF Aβ42/40 binary status, and T+/− indicates CSF p-tau181 binary status.

In Figure 2, we display predicted longitudinal cognitive trajectories according to baseline CSF AT profiles. In the ADNI cohort, A−T+ individuals did not present with significantly different rates of decline in mPACC as compared with A−T− individuals, regardless of baseline cognitive status (CU: β = –0.09; 95% CI, −0.3 to 0.1; *P* = .27; MCI: β = −0.01; 95% CI, −0.4 to 0.4; *P* = .99). In the WISC cohort, which included predominantly CU individuals, the A−T+ profile did not show significantly different rates of decline in PACC-3 scores compared with A−T− individuals (β = −0.02; 95% CI, −0.05 to 0.02; *P* = .28). In both the ADNI (CU and MCI) and WISC (CU at baseline) cohorts, individuals with A+T− and A+T+ profiles had faster cognitive decline when compared with those with an A−T− profile (eTables 2 and 3 in Supplement 1). When using more stringent p-tau cutoff values, similar results were obtained. Full models from both main and sensitivity analyses are available in eTables 2, 3, 4, and 5 in Supplement 1.

### Change in Imaging Markers

**Figure 3.**
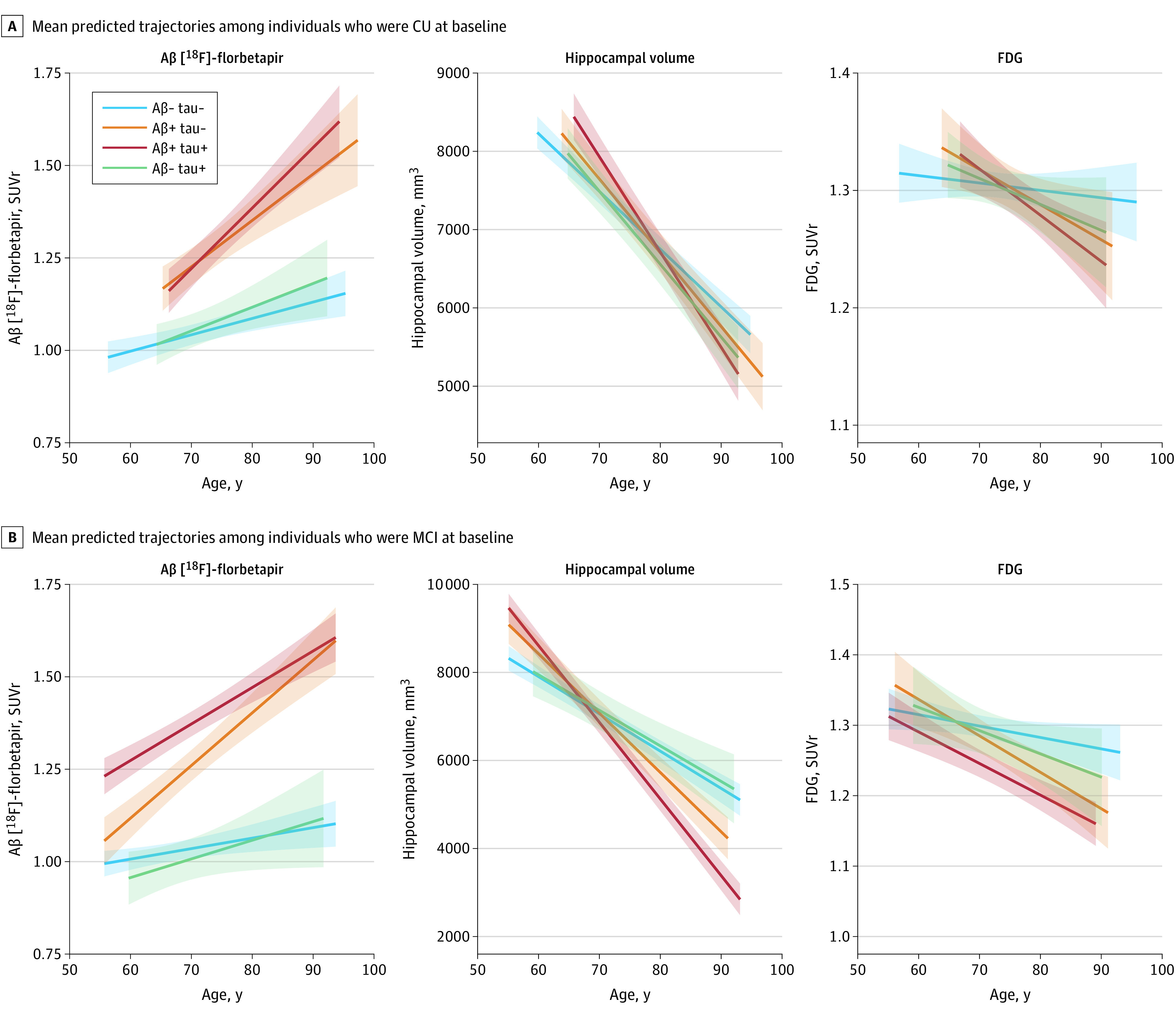
Associations Between Cerebrospinal Fluid (CSF) Amyloid-Tau (AT) Status and Longitudinal Biomarker Signs of β-Amyloid (Aβ) Accumulation and Neurodegeneration Mean predicted trajectories of Aβ [^18^F]-florbetapir and [^18^F]–fluorodeoxyglucose (FDG) positron emission tomography (PET), as well as magnetic resonance imaging (MRI)–derived hippocampal volume according to CSF AT status (only in Alzheimer’s Disease Neuroimaging Initiative [ADNI]) are shown for individuals who were cognitively unimpaired (CU; A) or had mild cognitive impairment (MCI; B) at baseline. The mean predicted trajectories for the Aβ-PET and FDG-PET, as well as hippocampal volume are displayed with solid lines and 95% CIs. Trajectories were estimated including terms for CSF AT status, covariates (age, years of education, *APOE* ε4 genotype, sex), and the age × CSF AT status interaction. A+/− indicates CSF Aβ42/40 binary status, and T+/− indicates CSF p-tau181 binary status.

Next, longitudinal imaging biomarkers were used to assess disease progression over time (Figure 3). In the ADNI cohort, regardless of their baseline cognitive status, individuals with an A−T+ profile did not differ significantly from those with A−T− in longitudinal rates of change in Aβ-PET (CU: β = 0.002; 95% CI, −0.01 to 0.01; *P* = .44; MCI: β = 0.002; 95% CI, −0.01 to 0.01; *P* = .44), FDG-PET (CU: β = −0.002; 95% CI, −0.01 to 0.001; *P* = .28, MCI: β = −0.002; 95% CI, −0.005 to 0.002; *P* = .39), or hippocampal volume (CU: β = −19 mm^3^ per year; 95% CI, −40 to 1.0; *P* = .06; MCI: β = 3.9 mm^3^ per year; 95% CI, −29 to 37; *P* = .82), although numerically lower in CU individuals. In contrast and in accordance with previous studies, individuals with MCI and A+T+ or A+T− profiles, when compared with those with an A−T− profile, displayed significant changes in markers of hippocampal volume and glucose metabolism (FDG PET and MRI, respectively) (eTables 6 and 7 in Supplement 1). In CU with an A+T− profile, no significant changes were seen in change in FDG PET, whereas there was a slight decrease in hippocampal volume as compared with individuals with an A−T− profile (eTables 6 and 7 in Supplement 1). However, significant time-related increases in Aβ-PET were seen for individuals with both A+T− and A+T+ profiles independent of baseline cognitive status. Changing the cutoff values for p-tau yielded very similar results. Full models from both main and sensitivity analyses are available in eTables 6, 7, 8, 9, 10, and 11 in Supplement 1.

### Fibrillar Tau Deposition

**Figure 4.**
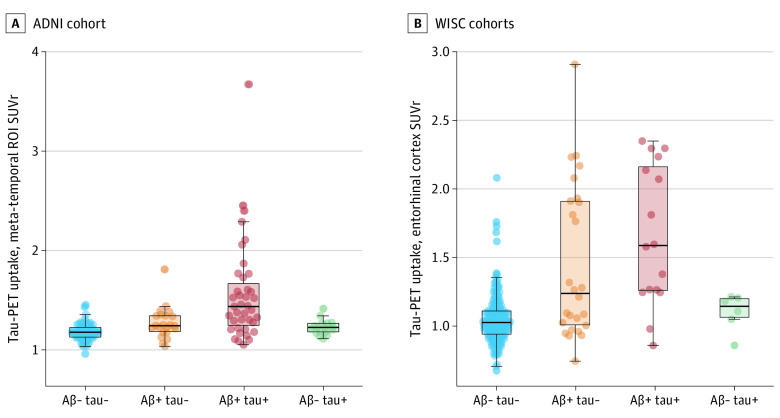
Cross-Sectional Differences in Tau Positron Emission Tomography (PET) Deposition by Cerebrospinal Fluid (CSF) Amyloid-Tau (AT) Status Cross-sectional analysis examining differences in tau-PET uptake across CSF AT groups using [^18^F]-flortaucipir in Alzheimer’s Disease Neuroimaging Initiative (ADNI) (A) and [^18^F]-MK6240 in 2 Wisconsin cohorts, Wisconsin Alzheimer Disease Research Center and Wisconsin Registry for Alzheimer Prevention (WISC) (B). Linear models, including age, sex, *APOE*ε4 status, and years of education as covariates were used in both cohorts. A+/− indicates CSF Aβ42/40 binary status, and T+/− indicates CSF p-tau181 binary status.

In a subset of participants who had tau-PET data (ADNI: n = 192; WISC: n = 227), we evaluated whether isolated p-tau positivity was associated with the deposition of fibrillar tau (Figure 4). In the ADNI cohort, individuals with available tau-PET in the A−T+ CSF group did not present statistically significant differences in [^18^F]-flortaucipir meta-temporal ROI standardized uptake value ratio (SUVr) values (estimated marginal mean [EM] = 1.2; SE = 0.06) in comparison with the A−T− group (*P* = .40) (EM = 1.2; SE = 0.03). In the WISC cohort, the A−T+ group (EM = 1.1; SE = 0.12) did not show significantly different [^18^F]-MK6240 tau-PET SUVr than the A−T− group (EM = 1.1; SE = 0.20; *P* = .99). In both the ADNI and WISC cohorts, the A+T+ group had higher tau PET SUVr (ADNI: EM = 1.54; SE = 0.03; *P* < .001; WISC: EM = 1.5; SE = 0.04; *P* = .002) compared with the A−T+ group. Differences were less clear for the CSF A+T−group (eTables 12 and 13 in Supplement 1).

## Discussion

In this cohort study, we found that the prevalence of CSF A−T+ in a clinical neurochemistry laboratory, likely to be representative of a general memory clinic population, was 4.1%, which is far lower than the prevalence of other CSF profiles. Further, compared with the A−T− biomarker profile, we found that the A−T+ profile was not associated with higher rates of cognitive deterioration, Aβ accumulation, tau PET pathology, or neurodegeneration.

Several studies have attempted to determine the prevalence of AD pathophysiology in varying scenarios. We found that in the UGOT cohort, CSF Aβ abnormalities ranged from approximately 25% in the youngest individuals to roughly 60% in the oldest individuals. These estimates are lower than those found in a recently published, large, multicenter study evaluating the prevalence of Aβ positivity as determined by either PET or CSF.^33^ This slight discrepancy may be explained by the presence of other disorders causing cognitive symptoms in our cohort and likely reflects more similarly what may be expected in a real-world memory clinic, considering most CSF analyses are ordered by memory clinics or general neurology centers across Sweden. However, fewer studies have performed in-depth evaluations of the clinical meaning of CSF results not consistent with the current view of the chain of AD pathophysiological events. Previous studies evaluating the prevalence of CSF AT abnormalities report mixed results. Although many studies report higher figures (above approximately 15%) for the prevalence of an A−T+ profile, most of these have relied on CSF Aβ42 alone to define “A” status.^7,9,10,34,35^ When defining “A” with the widely recommended Aβ42/40 ratio,^36^ this prevalence is substantially reduced to figures like the one reported here for this large, unselected clinical chemistry cohort (approximately 4%).^8^ This suggests that the absence of correction for Aβ40 concentrations may lead to artificially deflated frequencies for CSF Aβ abnormalities, and, consequently, artificially inflated frequencies for a CSF A−T+ profile. This highlights the need for using CSF Aβ42/40 in clinical settings when separately assessing “A” and “T” CSF biomarkers.

Furthermore, discussing the etiology underlying a CSF A−T+ profile is highly needed. For instance, individuals presenting with this profile are sometimes referred to as suspected non-Alzheimers pathology. This construct is most often biomarker defined as Aβ negativity in the presence of neurodegeneration and a clinical picture not clearly consistent with a recognizable non-AD phenotype.^6^ More specifically, during the early phases of AD CSF biomarker research, p-tau was considered by some studies as a biomarker of neuronal injury.^10^ However, it is now known that biomarker abnormalities, especially in “T” and “N” (for neurodegeneration) categories, do not provide the same information within and between modalities.^35^ Some authors argue that a CSF A−T+ pattern may reflect tau pathophysiology not attributable to Aβ accumulation, such as primary age-related taupathy, but this theory has become increasingly untenable due to the accumulation of fluid biomarker evidence that tangle pathology in the absence of concurrent Aβ pathology is not well reflected by p-tau,^37,38^ although data specifically addressing this question are still scarce. Importantly, we found that the A−T+ group had similar tracer retention in temporal areas as the A−T− group, suggesting that an isolated elevation of CSF p-tau is not due to fibrillar tau pathology in the absence of Aβ pathology. However, it is important to note that to date, no convincing evidence has shown that tau-PET tracers are capable of capturing PART, which remains a lingering question for the field.

In this challenging context of determining the underpinnings of a CSF A−T+ profile, a potentially better-suited explanation is that the finding of increased p-tau in the absence of brain amyloidosis reflects slower CSF turnover. As an example of how alterations in CSF dynamics can impact biomarker readings, low concentrations of all core biomarkers (ie, p-tau, t-tau, Aβ42, and Aβ40) are commonly seen in cases of idiopathic normal pressure hydrocephalus, not necessarily associated with AD pathophysiology.^39^ Interestingly, in the UGOT and ADNI cohorts, concentrations of Aβ40 and Aβ42 were clearly higher in the A−T+ group, compared with the A−T− group. This could potentially suggest that the CSF A−T+ profile is indeed associated altered CSF dynamics, given that within 2 Aβ-negative groups as defined by the Aβ42/Aβ40 ratio, it would not be expected that Aβ42 and Aβ40 values alone would differ between groups.

Data from both longitudinal cohorts suggest that individuals with an A−T+ pattern had similar cognitive and biomarker trajectories as compared with participants with a CSF A−T− profile. This aligns with recent findings in individuals with subjective cognitive decline that showed that the A−T+ group was not more likely to progress to MCI or dementia compared with the biomarker negative group when defining “A” using Aβ PET or Aβ42 and “T” using p-tau.^7,40^ However, when defining A−T+ with both Aβ− and tau-PET, individuals with this profile show slightly greater risk of cognitive decline than biomarker-negative individuals.^41^

Lastly, the choice of cutoff values may influence the interpretation of abnormal results. For Aβ42/40, this is likely of limited relevance because it usually presents a bimodal distribution^23^ and concordance with amyloid PET usually reaches over 90%,^42^ which in turn makes cutoff determination and interpretation uncomplicated. However, p-tau typically shows no such distribution and is less clearly linked with tau-PET status.^43^ Here, we show that both p-tau cutoff values that optimize sensitivity and specificity, as well as higher, less sensitive cutoff values presented very similar results in our study.

### Strengths and Limitations

Strengths of this study include the large sample size of participants with core CSF biomarkers of Aβ and soluble tau pathology, allowing us to derive estimated prevalence of these pathologies in a real-world, unselected clinical routine sample, for whom memory clinic physicians order most CSF tests. Further, our consistent finding that CSF A−T+ individuals do not show significant cognitive decline suggests that the findings are generalizable across different populations. Limitations include limited clinical information from the UGOT cohort, as the data are derived from a database used in laboratory practice. This precludes us from defining strict inclusion/exclusion criteria and describing the clinical features of the patients included. Another potential limitation is that the longitudinal cohorts consisted primarily of self-identified White individuals. Finally, AT status in the WISC sample was based on CSF tests not necessarily coinciding with the cognitive baseline visit.

## Conclusions

In this cohort study, we found the estimated prevalence of a CSF A−T+ profile to be 4.1% in a large, real-world, clinical routine data set. Further, individuals with this profile did not present with significantly higher rates of cognitive decline or AD biomarker progression than those of individuals with an A−T− profile. We suggest that practitioners encountering this pattern in daily clinical practice should interpret such a finding similarly to CSF biomarker-negative results during the diagnostic workup of patients with cognitive complaints, and unusually high CSF Aβ42 and Aβ40 concentrations could be a recognizable feature of such a profile.

## Notes

Supplement 1. **eMethods 1.** UGOT, ADNI, and WISC **eMethods 2.** CSF Handling and Assays in UGOT, ADNI, and WISC **eMethods 3.** Imaging Acquisition and Processing in ADNI and WISC **eMethods 4.** Cognitive Composites in ADNI and WISC **eMethods 5.** Greyscale and Drift Analyses in the UGOT Data Set **eTable 1.** Greyscale Analyses in the UGOT Data Set **eTable 2.** Linear Mixed-Effects Models of Baseline CSF AT Profiles and Longitudinal mPACC in CU and MCI Individuals in ADNI **eTable 3.** Linear Mixed-Effects Models of Baseline CSF AT Profiles and Longitudinal PACC-3 in WISC **eTable 4.** Sensitivity Analysis Using Linear Mixed-Effects Models of Baseline CSF AT Profiles and Longitudinal mPACC in CU And MCI Individuals In ADNI Using 15% Higher P-Tau Cutoffs **eTable 5.** Sensitivity Analysis Using Linear Mixed-Effects Models of Baseline CSF AT Profiles and Longitudinal PACC-3 in WISC Using 15% Higher P-Tau cutoffs **eTable 6.** Linear Mixed-Effects Models of Baseline CSF AT Profiles and Longitudinal FDG PET in CU and MCI Individuals in ADNI **eTable 7.** Linear Mixed-Effects Models of Baseline CSF AT Profiles and Longitudinal MRI in CU and MCI Individuals in ADNI **eTable 8.** Linear Mixed-Effects Models of Baseline CSF AT Profiles and Longitudinal Aβ PET in CU and MCI Individuals in ADNI **eTable 9.** Sensitivity Analysis Using Linear Mixed-Effects Models of Baseline CSF AT Profiles and Longitudinal Aβ PET in CU and MCI Individuals in ADNI Using 15% Higher P-Tau Cutoffs **eTable 10.** Sensitivity Analysis Using Linear Mixed-Effects Models of Baseline CSF AT Profiles and Longitudinal FDG PET in CU and MCI Individuals in ADNI Using 15% Higher P-Tau Cutoffs **eTable 11.** Sensitivity Analysis Using Linear Mixed-Effects Models of Baseline CSF AT Profiles and Longitudinal MRI in CU and MCI Individuals in ADNI Using 15% Higher P-Tau Cutoffs **eTable 12.** Cross-Sectional Tau PET Analysis in ADNI **eTable 13.** Cross-Sectional Tau PET Analysis in WISC **eFigure 1.** Age-Stratified Prevalence of CSF AT Profiles Using CSF Aβ42 Alone for “A” **eFigure 2.** Drift Analyses in the UGOT Data Set
eReferences

Supplement 2. **Nonauthor Collaborators.** ADNI Cohort.

Supplement 3.
Data Sharing Statement
