# Supplementary material for: Prevalence and Clinical Implications of a β-Amyloid–Negative, Tau-Positive Cerebrospinal Fluid Biomarker Profile in Alzheimer Disease
Source: JAMA Neurol. 2023 Jul 31;80(9):969–79. doi: 10.1001/jamaneurol.2023.2338 (PMC10391361; doi:10.1001/jamaneurol.2023.2338)
Supplement: Supplement 1. — eMethods 1. UGOT, ADNI, and WISC eMethods 2. CSF Handling and Assays in UGOT, ADNI, and WISC eMethods 3. Imaging Acquisition and Processing in ADNI and WISC eMethods 4. Cognitive Composites in ADNI and WISC eMethods 5. Greyscale and Drift Analyses in the UGOT Data Set eTable 1. Greyscale Analyses in the UGOT Data Set eTable 2. Linear Mixed-Effects Models of Baseline CSF AT Profiles and Longitudinal mPACC in CU and MCI Individuals in ADNI eTable 3. Linear Mixed-Effects Models of Baseline CSF AT Profiles and Longitudinal PACC-3 in WISC eTable 4. Sensitivity Analysis Using Linear Mixed-Effects Models of Baseline CSF AT Profiles and Longitudinal mPACC in CU And MCI Individuals In ADNI Using 15% Higher P-Tau Cutoffs eTable 5. Sensitivity Analysis Using Linear Mixed-Effects Models of Baseline CSF AT Profiles and Longitudinal PACC-3 in WISC Using 15% Higher P-Tau cutoffs eTable 6. Linear Mixed-Effects Models of Baseline CSF AT Profiles and Longitudinal FDG PET in CU and MCI Individuals in ADNI eTable 7. Linear Mixed-Effects Models of Baseline CSF AT Profiles and Longitudinal MRI in CU and MCI Individuals in ADNI eTable 8. Linear Mixed-Effects Models of Baseline CSF AT Profiles and Longitudinal Aβ PET in CU and MCI Individuals in ADNI eTable 9. Sensitivity Analysis Using Linear Mixed-Effects Models of Baseline CSF AT Profiles and Longitudinal Aβ PET in CU and MCI Individuals in ADNI Using 15% Higher P-Tau Cutoffs eTable 10. Sensitivity Analysis Using Linear Mixed-Effects Models of Baseline CSF AT Profiles and Longitudinal FDG PET in CU and MCI Individuals in ADNI Using 15% Higher P-Tau Cutoffs eTable 11. Sensitivity Analysis Using Linear Mixed-Effects Models of Baseline CSF AT Profiles and Longitudinal MRI in CU and MCI Individuals in ADNI Using 15% Higher P-Tau Cutoffs eTable 12. Cross-Sectional Tau PET Analysis in ADNI eTable 13. Cross-Sectional Tau PET Analysis in WISC eFigure 1. Age-Stratified Prevalence of CSF AT Profiles Using CSF Aβ42 Alone for “A” eFigure 2. Drift A [file jamaneurol-e232338-s001.pdf]

Supplementary Online Content

Erickson P, Simrén J, Brum WS, et al; ADNI Cohort. Prevalence and clinical implications of a β-amyloid–negative, tau-positive cerebrospinal fluid biomarker profile in Alzheimer disease. *JAMA Neurol*. Published online July 31, 2023. doi:10.1001/jamaneurol.2023.2338

eMethods 1. UGOT, ADNI, and WISC

eMethods 2. CSF Handling and Assays in UGOT, ADNI, and WISC

eMethods 3. Imaging Acquisition and Processing in ADNI and WISC

eMethods 4. Cognitive Composites in ADNI and WISC

eMethods 5. Greyscale and Drift Analyses in the UGOT Data Set

eTable 1. Greyscale Analyses in the UGOT Data Set

eTable 2. Linear Mixed-Effects Models of Baseline CSF AT Profiles and Longitudinal mPACC in CU and MCI Individuals in ADNI

eTable 3. Linear Mixed-Effects Models of Baseline CSF AT Profiles and Longitudinal PACC-3 in WISC

eTable 4. Sensitivity Analysis Using Linear Mixed-Effects Models of Baseline CSF AT Profiles and Longitudinal mPACC in CU And MCI Individuals In ADNI Using 15% Higher P-Tau Cutoffs

eTable 5. Sensitivity Analysis Using Linear Mixed-Effects Models of Baseline CSF AT Profiles and Longitudinal PACC-3 in WISC Using 15% Higher P-Tau cutoffs

eTable 6. Linear Mixed-Effects Models of Baseline CSF AT Profiles and Longitudinal FDG PET in CU and MCI Individuals in ADNI

eTable 7. Linear Mixed-Effects Models of Baseline CSF AT Profiles and Longitudinal MRI in CU and MCI Individuals in ADNI

eTable 8. Linear Mixed-Effects Models of Baseline CSF AT Profiles and Longitudinal Aβ PET in CU and MCI Individuals in ADNI

eTable 9. Sensitivity Analysis Using Linear Mixed-Effects Models of Baseline CSF AT Profiles and Longitudinal Aβ PET in CU and MCI Individuals in ADNI Using 15% Higher P-Tau Cutoffs

eTable 10. Sensitivity Analysis Using Linear Mixed-Effects Models of Baseline CSF AT Profiles and Longitudinal FDG PET in CU and MCI Individuals in ADNI Using 15% Higher P-Tau Cutoffs

eTable 11. Sensitivity Analysis Using Linear Mixed-Effects Models of Baseline CSF AT Profiles and Longitudinal MRI in CU and MCI Individuals in ADNI Using 15% Higher P-Tau Cutoffs

eTable 12. Cross-Sectional Tau PET Analysis in ADNI

eTable 13. Cross-Sectional Tau PET Analysis in WISC

eFigure 1. Age-Stratified Prevalence of CSF AT Profiles Using CSF Aβ42 Alone for “A”

eFigure 2. Drift Analyses in the UGOT Data Set

eReferences

This supplementary material has been provided by the authors to give readers additional information about their work.

© 2023 Erickson P et al. *JAMA Neurology.*

eMethods 1. UGOT, ADNI, and WISC information.

UGOT

The University of Gothenburg cohort is based on clinical laboratory routine data extracted from the local database at the neurochemistry laboratory at Sahlgrenska University Hospital, Mölndal, Sweden. In this study, we included all participants over the age of 50 with data on Aβ42/40 and p-tau from the same time of collection between 7th of November 2019 and 18th of January 2021. All participants had data on age and biomarker concentrations, while clinical information on diagnosis and cognitive function was not available, as memory and neurology clinics send their referrals without accompanying clinical data. However, they are encouraged to send referrals for these analyses only for individuals above the age of 50 where there is a suspicion of a cognitive disorder.

ADNI

Data used in the preparation of this article were obtained from the Alzheimer's Disease Neuroimaging Initiative (ADNI) database (adni.loni.usc.edu), which is an ongoing longitudinal observational study. The ADNI was launched in 2003 as a public-private partnership, led by Principal Investigator Michael W. Weiner, MD. The primary goal of ADNI has been to test whether serial magnetic resonance imaging (MRI), positron emission tomography (PET), other biological markers, and clinical and neuropsychological assessment can be combined to measure the progression of mild cognitive impairment (MCI) and early Alzheimer's disease (AD). For up-to-date information, see www.adni-info.org.

WISC

The Wisconsin Registry for Alzheimer's Prevention (WRAP) is an ongoing longitudinal observational cohort study that was initiated in 2001. It is enriched with people with a parental history of probable AD dementia. Recruitment sources included memory clinics in which a parent was diagnosed or treated, limited radio and newspaper advertisements, community outreach events, and word of mouth. Participants generally meet the following inclusion criteria at study entry: age 40–65 years; fluent English speaker; visual and auditory acuity adequate for neuropsychological testing; good health with no diseases expected to interfere with study participation over time. Participants are excluded from enrollment if they have a prior diagnosis of dementia or evidence of dementia at baseline testing.

Longitudinal data collection for the Wisconsin Alzheimer's Disease Research Center (WI-ADRC) began in 2009 and is ongoing. Participants are recruited from Wisconsin Alzheimer's Institute-Affiliated Dementia Diagnostic Clinics, via community outreach events and lectures, media advertising, community partner organizations, and word of mouth. The cohort is enriched with middle-aged to older adult participants who have a parental history of probable AD and include participants across the clinical and biologic AD continuum.

© 2023 Erickson P et al. *JAMA Neurology.*

eMethods 2. CSF handling and assays in UGOT, ADNI and WISC.

UGOT

Cerebrospinal fluid (CSF) was collected at the referring center using standard procedures for lumbar punctures (LP) and was then collected in a 1.5 mL polypropylene low-binding tube, according to recent recommendations.[1] Next, the tube was transported at room temperature to the neurochemistry laboratory at Sahlgrenska University Hospital, Mölndal, Sweden within 24 hours of collection. Otherwise, the sample was partitioned in aliquots and transported frozen on dry ice, in accordance with international recommendations.[1] Previously published cut-offs were applied, determining an abnormal Aβ42/40 status when ≤0.072 using gaussian mixed modeling (GMM) and ≥50.2 pg/mL for p-tau using receiver operating characteristics (ROC) to best separate AD vs. controls.[2]

ADNI

Up to date methods on CSF collection in ADNI can be found elsewhere (https://adni.loni.usc.edu/methods/). In ADNI, a liquid chromatography mass spectrometry-based assay was used to determine the CSF Aβ42/40 ratio, whereas a fully automated Elecsys® assay (Roche Diagnostics International Ltd, Rotkreuz, Switzerland) was used to measure CSF p-tau. Briefly, the CSF handling was in accordance with recent international guidelines found elsewhere.[1] Previously published ADNI cutoffs of ≤ 0.0138 (using GMM) and ≥24 pg/mL (using a method maximizing identification of progressors from MCI to dementia) were used for determining abnormality and CSF Aβ42/40 and p-tau, respectively.[3,4]

In ADNI, the baseline visit comprised both LP and cognitive testing for all individuals.

WISC

CSF samples are collected in the core WRAP and WI-ADRC studies as well as linked studies. A center-wide standard pre-analytical protocol was used to collect approximately 22 mL of CSF that is subsequently gently mixed to remove collection gradients, partitioned into 0.5-mL aliquots in -1.5-mL polypropylene tubes, and stored at −80°C. CSF Aβ42, Aβ40 (research use only), and p-tau were quantified using the fully automated Elecsys® assays (Roche Diagnostics International Ltd, Rotkreuz, Switzerland). Abnormal status was determined as ≤ .046 for Aβ42/Aβ40 (using ROC-based classification with Aβ PET as the diagnostic standard) and ≥ 24.8 pg/mL (+2 SD above Aβ42/Aβ40 negative participants) for p-tau. More details on methods used to determine positivity can be found elsewhere.[5]

CSF AT status was determined from the baseline LP, which was not uniformly performed at the first cognitive visit. Baseline cognitive testing occurred on average 2.1 (SD = 2.8) years prior to baseline LP (median = 0.47 years prior; interquartile range = 2.90 years prior – 0.18 years prior; full range: 11.31 years prior – 2.47 years after). The last cognitive assessment occurred on average 4.8 (SD = 2.9) years after baseline LP (median = 5.6 years after; interquartile range: 2.14 years after – 7.02 years after; full range: 1.45 years prior – 10.23 years after). The A-T+ group did not differ from other status groups on time interval between baseline LP and baseline or final cognitive assessments.

© 2023 Erickson P et al. *JAMA Neurology.*

eMethods 3. Imaging acquisition and pre-processing methods.

ADNI

A global cortical composite normalizing to cerebellar grey matter was used for Aβ- and tau-PET.[6,7] The composite [18F]-florbetapir standard uptake value ratio (SUVr) value was generated for each participant by using the average SUVRs from the precuneus, prefrontal, orbitofrontal, parietal, temporal, anterior, and posterior cingulate cortices. A validated global composite was used for FDG-PET.[7] Hippocampal volume was normalized to total intracranial volume.[8] Further details on these widely used ADNI measures and its associated processing pipelines have been previously described.[7,8] The [^{18}F]-flortaucipir tau-PET meta-ROI composite SUVR was generated for each participant by using the average SUVRs from the entorhinal, amygdala, fusiform, inferior and middle temporal cortices, as previously described.[6]

WISC

[^{18}F]-MK6240 was used to quantify aggregated tau using previously established methods.[8] Briefly, the inferior cerebellum was used as reference, with SUVr calculated for the average uptake in the left and right entorhinal cortex. CSF AT status for the tau PET analysis was determined from the same LP as used for the cognitive trajectory analysis. That LP occurred on average 7.1 (SD = 2.9) years prior to tau PET. The time between tau-PET scan and LP did not differ between CSF AT groups.

© 2023 Erickson P et al. *JAMA Neurology.*

eMethods 4. Cognitive composites in ADNI and WISC cohorts.

To enable longitudinal analyses of the cognitive trajectories in this study, cognitive tests were used to generate standardized preclinical Alzheimer's cognitive composite (PACC) z-scores.

ADNI

In ADNI, we modeled the PACC z-scores at each timepoint as a function of age as outcome. However, the composite is instead made up by the Alzheimer's Disease Assessment scale, which constitutes the Cognitive Subscale Delayed Word Recall, Logical Memory Delayed Recall, MMSE, and (log-transformed) Trail-Making Test B Time to Completion; thus, creating the modified PACC (mPACC).[9,10]

In ADNI, individuals with MCI had a mean [SD] follow up of 4.61 [2.83] years, whereas CU individuals were followed for 6.02 [3.55] years.

WISC

In the WISC sample, three cognitive tests were used (thus generating the PACC-3): Total Recall score from Rey auditory verbal learning test (RAVLT), Trail Making Test Part B, and Logical Memory IIa (from the Wechsler Memory Scale)[10]. Following implementation of the National Alzheimer's Coordinating Center (NACC) Uniform Data Set (UDS) version 3, the WI-ADRC switched to using Craft story as the measure of story recall, thus a published crosswalk[9] was used to estimate Logical Memory IIa for those observations.[11] PACC-3 scores were standardized using the mean and standard deviation from the first cognitive assessment of cognitively unimpaired participants in the WRAP and WI-ADRC cohorts.

In WISC, there was an average [SD] of 4.9 [1.8] longitudinal PACC-3 scores. CSF AT status groups differed in the number of PACC-3 scores ($F=6.0$, $p = .001$). A+T+ and A-T+ groups had on average more longitudinal PACC-3 assessments, 5.7 [1.7] and 5.9 [2.1], respectively, than the A-T- group (Mean = 4.8 [1.8]). Of the 503 participants who had > 1 pacc-3 score, testing occurred over an average interval of 7.1 [2.9] years (range = 0.9 – 12.8).

© 2023 Erickson P et al. *JAMA Neurology.*

eMethods 5. Greyscale and drift analyses in the UGOT dataset.

To increase the robustness of our results to potential analytical and classification errors, we performed a drift and a grey-scale analysis. The drift analysis examined the influence of measurement abnormalities on our prevalence results and investigated the analytical performance for the Lumipulse instrument over time. The time frame 071119-181021 investigated was then divided into three equal episodes (A, B and C) as stated in eFigure 1. Subsequently, the three episodes were all internally compared with paired t-tests. Then, a grey-scale analysis was performed, showing the potential effects the present cut-offs might have on the prevalence outcome.

To accommodate this, the cut-offs for p-tau and Aβ42/40 were increased and lowered 15 %, respectively. Individuals having values deviating up to 15% above the cut-off value for p-tau and below the cut-off value for Aβ42/40 were then excluded, whereafter the prevalence was computed again. The cut-offs used in the greyscale analysis were Aβ42/40 ≥0.61 and p-tau ≤58 ng/L. We thus increased the thresholds of pathology and decreased the sensitivity of the cut-offs. Subsequently, it was compared to the initial prevalence analysis. The results are found in eTable 1.

© 2023 Erickson P et al. *JAMA Neurology.*

eTable 1. Greyscale analysis in the UGOT dataset.

	All	A-T-	A-T+	A+T-	A+T+
Current cut-offs, No. (%)	7679 (100)	3241 (42)	316 (4)	1324 (17)	2798 (36)
15 % less sensitive cut-offs, No. (%)	7679 (100)	3892 (51)	273 (4)	1218 (16)	2296 (30)
Individuals in grey zone excluded, No. (%)	4900 (100)	2400 (49)	148 (3)	497 (10)	1855 (38)

Grey-scale analysis was performed so that cut-offs for p-tau and Aβ42/40 were increased and lowered 15 %, respectively. Individuals having values deviating up to 15% above the cut-off value for p-tau and below the cut-off value for Aβ42/40 were then excluded, whereafter the prevalence was computed again. The cut-offs used in the greyscale analysis were Aβ42/40 ≥0.61 and p-tau ≤58 ng/L, whereas the original cut-offs were ≤0.072 for Aβ42/40 and ≥50.2 pg/mL for p-tau.

© 2023 Erickson P et al. *JAMA Neurology.*

eTable2. Linear mixed effects models of baseline CSF AT profiles and longitudinal mPACC in CU and MCI individuals in ADNI.

CU					
**Terms**	**Estimate (β)**	**95% CI**		**Standard error**	**P-value**
(Intercept)	-3.959	-5.751	-2.166	0.915	<0.001
A+T-	0.215	-0.634	1.065	0.434	0.620
A+T+	0.566	-0.327	1.459	0.456	0.215
A-T+	0.114	-0.757	0.985	0.444	0.798
Age	-0.076	-0.160	0.008	0.043	0.077
Education	0.270	0.161	0.378	0.055	<0.001
Gender	-1.209	-1.790	-0.627	0.297	<0.001
Practice	0.304	0.226	0.381	0.040	<0.001
APOE Ɛ4	-0.677	-1.364	0.011	0.351	0.055
A+T-*Age	-0.228	-0.380	-0.076	0.077	0.003
A+T+*Age	-0.347	-0.494	-0.201	0.075	<0.001
A-T+*Age	-0.090	-0.250	0.069	0.081	0.267
Age*Practice	-0.043	-0.050	-0.035	0.004	<0.001
**MCI**					
**Terms**	**Estimate (β)**	**95% CI**		**Standard error**	**P-value**
(Intercept)	-4.948	-9.290	-0.605	2.216	0.026
A+T-	-2.589	-4.776	-0.401	1.116	0.021
A+T+	-5.319	-7.172	-3.467	0.945	<0.001
A-T+	-0.494	-3.725	2.738	1.649	0.765
Age	0.203	0.030	0.377	0.088	0.022
Education	0.162	-0.095	0.419	0.131	0.217
Gender	1.122	-0.307	2.552	0.729	0.125
Practice	-0.287	-0.373	-0.201	0.044	<0.001
APOE Ɛ4	-0.699	-2.279	0.881	0.806	0.387
A+T-*Age	-0.614	-0.891	-0.336	0.142	<0.001
A+T+*Age	-1.280	-1.495	-1.065	0.110	<0.001
A-T+*Age	-0.002	-0.399	0.395	-0.009	0.993
Age*Practice	-0.025	-0.032	-0.017	-6.258	<0.001

© 2023 Erickson P et al. *JAMA Neurology.*

eTable3. Linear mixed effects models of baseline CSF AT profiles and longitudinal PACC-3 in WISC.

CU					
**Terms**	**Estimate (β)**	**95% CI**		**Standard error**	**P-value**
(Intercept)	-1.439	-1.942	-0.935	0.257	<0.001
Age	0.008	-0.001	0.016	0.004	0.071
Sex (male)	-0.722	-0.874	-0.571	0.077	<0.001
Education	0.109	0.080	0.139	0.015	<0.001
Cohort	0.091	-0.054	0.236	0.074	0.219
APOE ε4 (carrier)	0.087	-0.062	0.235	0.076	0.251
Practice	0.038	-0.007	0.084	0.028	0.095
A+T+	0.058	-0.267	0.382	0.165	0.728
A-T+	-0.165	-0.565	0.235	0.204	0.418
A+T-	0.030	-0.234	0.294	0.134	0.821
A+T+*Age	-0.086	-0.114	-0.059	0.0142	<0.001
A-T+*Age	-0.019	-0.054	0.015	0.018	0.276
A+T-*Age	-0.045	-0.070	-0.020	0.013	0.001

© 2023 Erickson P et al. *JAMA Neurology.*

eTable4. Sensitivity analysis using linear mixed effects models of baseline CSF AT profiles and longitudinal mPACC in CU and MCI individuals in ADNI using 15% higher p-tau cut-offs.

CU					
**Terms**	**Estimate (β)**	**95% CI**		**Standard error**	**P-value**
(Intercept)	-3.951	-5.739	-2.163	0.912	<0.001
A+T-	0.145	-0.633	0.922	0.397	0.716
A+T+	1.013	0.020	2.007	0.507	0.047
A-T+	0.455	-0.692	1.603	0.585	0.437
Age	-0.085	-0.164	-0.005	0.041	0.038
Education	0.268	0.159	0.377	0.055	<0.001
Gender	-1.197	-1.779	-0.616	0.297	<0.001
Practice	0.307	0.229	0.384	0.040	<0.001
APOE Ɛ4	-0.719	-1.406	-0.031	0.351	0.041
A+T-*Age	-0.191	-0.327	-0.055	0.069	0.006
A+T+*Age	-0.439	-0.599	-0.279	0.082	<0.001
A-T+*Age	-0.135	-0.339	0.069	0.104	0.195
Age*Practice	-0.042	-0.050	-0.034	0.004	<0.001
**MCI**					
**Terms**	**Estimate (β)**	**95% CI**		**Standard error**	**P-value**
(Intercept)	-5.025	-9.298	-0.753	2.180	0.022
A+T-	-2.189	-4.143	-0.234	0.997	0.029
A+T+	-5.995	-7.857	-4.132	0.950	<0.001
A-T+	0.474	-4.217	5.164	2.393	0.843
Age	0.204	0.039	0.370	0.085	0.016
Education	0.163	-0.093	0.418	0.130	0.213
Gender	1.034	-0.383	2.452	0.723	0.154
Practice	-0.288	-0.373	-0.202	0.044	<0.001
APOE Ɛ4	-0.600	-2.167	0.967	0.799	0.453
A+T-*Age	-0.687	-0.930	-0.444	0.124	<0.001
A+T+*Age	-1.356	-1.573	-1.138	0.111	<0.001
A-T+*Age	-0.017	-0.579	0.544	0.287	0.952
Age*Practice	-0.025	-0.032	-0.017	0.004	<0.001

© 2023 Erickson P et al. *JAMA Neurology.*

eTable5. Sensitivity analysis using linear mixed effects models of baseline CSF AT profiles and longitudinal PACC-3 in WISC using 15% higher p-tau cut-offs.

CU					
**Terms**	**Estimate (β)**	**95% CI**		**Standard error**	**P-value**
(Intercept)	-1.422	-1.926	-0.919	0.256	<.001
Age	0.007	-0.002	0.015	0.004	0.118
Sex (male)	-0.721	-0.873	-0.570	0.077	<.001
Education	0.108	0.079	0.138	0.015	<.001
Cohort	0.095	-0.050	0.240	0.074	0.198
APOE ε4	0.089	-0.059	0.237	0.075	0.240
Practice	0.037	-0.008	0.083	0.023	0.108
A+T+	0.298	-0.155	0.751	0.231	0.197
A-T+	-0.331	-0.888	0.227	0.284	0.245
A+T-	-0.019	-0.252	0.214	0.119	0.875
A+T+*Age	-0.096	-0.131	-0.060	0.018	<.001
A-T+*Age	0.002	-0.045	0.049	0.024	0.947
A+T-*Age	-0.050	-0.073	-0.028	0.012	<.001

© 2023 Erickson P et al. *JAMA Neurology.*

eTable6. Linear mixed effects models of baseline CSF AT profiles and longitudinal Aβ PET in CU and MCI individuals in ADNI.

CU					
**Terms**	**Estimate (β)**	**95% CI**		**Standard error**	**P-value**
(Intercept)	1.012	0.907	1.117	0.054	<0.001
A+T-	0.211	0.161	0.260	0.025	<0.001
A+T+	0.219	0.170	0.267	0.025	<0.001
A-T+	0.017	-0.030	0.065	0.024	0.482
Age	0.004	0.002	0.007	0.001	<0.001
Education	0.003	-0.004	0.009	0.003	0.399
Gender	-0.059	-0.092	-0.027	0.017	<0.001
APOE Ɛ4	0.050	0.013	0.088	0.019	0.009
A+T-*Age	0.008	0.003	0.013	0.003	0.003
A+T+*Age	0.012	0.007	0.017	0.002	<0.001
A-T+*Age	0.002	-0.003	0.007	0.003	0.436
**MCI**					
**Terms**	**Estimate (β)**	**95% CI**		**Standard error**	**P-value**
(Intercept)	1.008	0.924	1.093	0.043	<0.001
A+T-	0.244	0.203	0.286	0.021	<0.001
A+T+	0.349	0.315	0.383	0.017	<0.001
A-T+	-0.024	-0.084	0.035	0.030	0.426
Age	0.003	0.001	0.005	0.001	0.009
Education	0.002	-0.003	0.007	0.003	0.446
Gender	-0.028	-0.055	-0.001	0.014	0.040
APOE Ɛ4	0.051	0.022	0.080	0.015	0.001
A+T-*Age	0.011	0.007	0.015	0.002	<0.001
A+T+*Age	0.007	0.004	0.010	0.002	<0.001
A-T+*Age	0.002	-0.003	0.008	0.003	0.437

For CU and MCI individuals, the profiles A-T-, A+T-, A+T+ and A-T+, constituted 159, 44, 53 and 40, in addition to 171, 77, 177 and 27 individuals, respectively. CU individuals had a mean [SD] 3.05 [1.45] scans, whereas MCI individuals had 2.57 [1.34] scans.

© 2023 Erickson P et al. *JAMA Neurology.*

eTable7. Linear mixed effects models of baseline CSF AT profiles and longitudinal FDG PET in CU and MCI individuals in ADNI.

CU					
**Terms**	**Estimate (β)**	**95% CI**		**Standard error**	**P-value**
(Intercept)	1.299	1.261	1.338	0.020	<0.001
A+T-	0.002	-0.016	0.021	0.010	0.796
A+T+	-0.001	-0.019	0.018	0.009	0.942
A-T+	-0.002	-0.020	0.016	0.009	0.816
Age	-0.001	-0.002	0.001	0.001	0.378
Education	0.000	-0.002	0.003	0.001	0.809
Gender	-0.005	-0.017	0.007	0.006	0.424
APOE Ɛ4	-0.008	-0.023	0.007	0.008	0.289
A+T-*Age	-0.002	-0.005	0.001	0.001	0.107
A+T+*Age	-0.003	-0.006	-0.001	0.001	0.015
A-T+*Age	-0.002	-0.004	0.001	0.001	0.284
**MCI**					
**Terms**	**Estimate (β)**	**95% CI**		**Standard error**	**P-value**
(Intercept)	1.317	1.270	1.364	0.024	<0.001
A+T-	-0.021	-0.044	0.001	0.012	0.065
A+T+	-0.059	-0.078	-0.040	0.010	<0.001
A-T+	-0.010	-0.042	0.022	0.016	0.541
Age	-0.002	-0.003	0.000	0.001	0.049
Education	-0.001	-0.004	0.001	0.001	0.348
Gender	0.010	-0.005	0.026	0.008	0.182
APOE Ɛ4	-0.025	-0.042	-0.008	0.009	0.004
A+T-*Age	-0.004	-0.006	-0.001	0.001	0.018
A+T+*Age	-0.003	-0.005	-0.001	0.001	0.012
A-T+*Age	-0.002	-0.005	0.002	0.002	0.387

For CU and MCI individuals, the profiles A-T-, A+T-, A+T+ and A-T+, constituted 176, 52, 63 and 45, in addition to 184, 90, 220 and 34 individuals, respectively. CU individuals had a mean [SD] 2.13 [1.63] scans, whereas MCI individuals had 2.42 [1.79] scans.

© 2023 Erickson P et al. *JAMA Neurology.*

eTable8. Linear mixed effects models of baseline CSF AT profiles and longitudinal MRI in CU and MCI individuals in ADNI.

CU					
**Terms**	**Estimate (β)**	**95% CI**		**Standard error**	**P-value**
(Intercept)	7603	7050	8157	282.3	7050
A+T-	79.66	-175.2	334.5	130.0	-175.2
A+T+	259.6	12.51	506.7	126.1	12.51
A-T+	-72.21	-339.0	194.6	136.1	-339.0
Age	-73.81	-83.03	-64.59	4.703	-83.03
Education	-24.35	-57.89	9.194	17.11	-57.89
Gender	529.3	353.4	705.2	89.75	353.4
APOE Ɛ4	-99.95	-306.4	106.5	105.3	-306.4
A+T-*Age	-20.40	-39.56	-1.234	9.777	-39.56
A+T+*Age	-47.91	-66.39	-29.43	9.428	-66.39
A-T+*Age	-19.38	-39.77	1.007	10.40	-39.77
**MCI**					
**Terms**	**Estimate (β)**	**95% CI**		**Standard error**	**P-value**
(Intercept)	7129	6514	7744	313.8	6514
A+T-	-87.82	-391.6	216.0	155.0	-391.6
A+T+	-378.9	-630.6	-127.1	128.4	-630.6
A-T+	88.58	-364.1	541.3	231.0	-364.1
Age	-84.65	-97.06	-72.23	6.333	-97.06
Education	-15.49	-52.28	21.31	18.77	-52.28
Gender	534.0	328.5	739.5	104.9	328.5
APOE Ɛ4	-294.8	-515.6	-73.96	112.7	-515.6
A+T-*Age	-50.16	-72.39	-27.93	11.34	-72.39
A+T+*Age	-89.54	-106.8	-72.28	8.805	-106.8
A-T+*Age	3.908	-28.82	36.64	16.70	-28.82

For CU and MCI individuals, the profiles A-T-, A+T-, A+T+ and A-T+, constituted 185, 60, 69 and 47, in addition to 198, 101, 255 and 33 individuals, respectively. CU individuals had a mean [SD] 4.61 [2.22] scans, whereas MCI individuals had 4.64 [2.10] scans.

© 2023 Erickson P et al. *JAMA Neurology.*

eTable9. Sensitivity analysis using linear mixed effects models of baseline CSF AT profiles and longitudinal Aβ PET in CU and MCI individuals in ADNI using 15% higher p-tau cut-offs.

CU					
**Terms**	**Estimate (β)**	**95% CI**		**Standard error**	**P-value**
(Intercept)	1.013	0.908	1.118	0.054	<0.001
A+T-	0.201	0.156	0.246	0.023	<0.001
A+T+	0.230	0.176	0.284	0.028	<0.001
A-T+	-0.001	-0.067	0.065	0.034	0.974
Age	0.004	0.002	0.006	0.001	<0.001
Education	0.003	-0.003	0.009	0.003	0.376
Gender	-0.059	-0.092	-0.027	0.017	<0.001
APOE Ɛ4	0.051	0.013	0.088	0.019	0.008
A+T-*Age	0.010	0.005	0.014	0.002	<0.001
A+T+*Age	0.011	0.006	0.016	0.003	<0.001
A-T+*Age	0.005	-0.001	0.012	0.003	0.107
**MCI**					
**Terms**	**Estimate (β)**	**95% CI**		**Standard error**	**P-value**
(Intercept)	0.993	0.908	1.078	0.043	<0.001
A+T-	0.259	0.221	0.296	0.019	<0.001
A+T+	0.363	0.327	0.398	0.018	<0.001
A-T+	-0.019	-0.103	0.065	0.043	0.664
Age	0.003	0.001	0.005	0.001	0.004
Education	0.003	-0.002	0.008	0.003	0.270
Gender	-0.032	-0.059	-0.005	0.014	0.021
APOE Ɛ4	0.054	0.024	0.083	0.015	<0.001
A+T-*Age	0.010	0.007	0.014	0.002	<0.001
A+T+*Age	0.006	0.003	0.010	0.002	<0.001
A-T+*Age	0.001	-0.007	0.008	0.004	0.830

For CU and MCI individuals, the profiles A-T-, A+T-, A+T+ and A-T+, constituted 180, 55, 42 and 19, in addition to 185, 107, 147 and 13 individuals, respectively.

© 2023 Erickson P et al. *JAMA Neurology.*

eTable10. Sensitivity analysis using linear mixed effects models of baseline CSF AT profiles and longitudinal FDG PET in CU and MCI individuals in ADNI using 15% higher p-tau cut-offs.

CU					
**Terms**	**Estimate (β)**	**95% CI**		**Standard error**	**P-value**
(Intercept)	1.298	1.260	1.336	0.019	<0.001
A+T-	-0.002	-0.019	0.015	0.009	0.793
A+T+	0.007	-0.014	0.028	0.011	0.499
A-T+	-0.011	-0.035	0.013	0.012	0.379
Age	-0.001	-0.002	0.001	0.001	0.348
Education	<0.001	-0.002	0.003	0.001	0.713
Gender	-0.006	-0.018	0.007	0.006	0.365
APOE Ɛ4	-0.008	-0.023	0.006	0.008	0.264
A+T-*Age	-0.001	-0.004	0.001	0.001	0.262
A+T+*Age	-0.005	-0.008	-0.002	0.001	0.001
A-T+*Age	-0.003	-0.007	0.001	0.002	0.134
**MCI**					
**Terms**	**Estimate (β)**	**95% CI**		**Standard error**	**P-value**
(Intercept)	1.317	1.271	1.364	0.024	<0.001
A+T-	-0.026	-0.046	-0.005	0.010	0.014
A+T+	-0.061	-0.081	-0.042	0.010	<0.001
A-T+	-0.001	-0.049	0.047	0.024	0.964
Age	-0.002	-0.003	<0.001	0.001	0.023
Education	-0.001	-0.004	0.001	0.001	0.305
Gender	0.011	-0.004	0.026	0.008	0.159
APOE Ɛ4	-0.025	-0.042	-0.008	0.009	0.004
A+T-*Age	-0.003	-0.006	-0.001	0.001	0.019
A+T+*Age	-0.003	-0.005	-0.001	0.001	0.016
A-T+*Age	-0.002	-0.007	0.003	0.003	0.461

For CU and MCI individuals, the profiles A-T-, A+T-, A+T+ and A-T+, constituted 199, 65, 50 and 22, in addition to 203, 128, 182 and 15 individuals, respectively.

© 2023 Erickson P et al. *JAMA Neurology.*

eTable11. Sensitivity analysis using linear mixed effects models of baseline CSF AT profiles and longitudinal MRI in CU and MCI individuals in ADNI using 15% higher p-tau cut-offs.

CU					
**Terms**	**Estimate (β)**	**95% CI**		**Standard error**	**P-value**
(Intercept)	7572	7020	8125	281.7	0.001
A+T-	141.9	-89.15	372.9	117.9	0.230
A+T+	234.6	-37.44	506.7	138.8	0.092
A-T+	-169.8	-524.9	185.4	181.2	0.349
Age	-75.95	-84.62	-67.28	4.423	0.001
Education	-22.37	-55.97	11.23	17.14	0.193
Gender	527.4	351.5	703.3	89.74	0.001
APOE Ɛ4	-96.19	-302.4	110.0	105.2	0.361
A+T-*Age	-18.74	-35.90	-1.567	8.760	0.033
A+T+*Age	-53.33	-73.67	-32.98	10.38	0.001
A-T+*Age	-15.4	-41.55	10.75	13.34	0.250
**MCI**					
**Terms**	**Estimate (β)**	**95% CI**		**Standard error**	**P-value**
(Intercept)	7136	6527	7745	310.7	<0.001
A+T-	-82.81	-354.9	189.3	138.8	0.551
A+T+	-412.2	-667.1	-157.4	130	0.002
A-T+	491.8	-178.1	1162	341.8	0.151
Age	-83.47	-95.4	-71.54	6.087	<0.001
Education	-16.95	-53.75	19.84	18.77	0.367
Gender	537.8	333.2	742.4	104.4	<0.001
APOE Ɛ4	-293	-512.8	-73.07	112.2	0.009
A+T-*Age	-57.57	-77.35	-37.79	10.09	<0.001
A+T+*Age	-93.7	-111.4	-75.97	9.047	<0.001
A-T+*Age	-12.01	-58.65	34.62	23.79	0.614

For CU and MCI individuals, the profiles A-T-, A+T-, A+T+ and A-T+, constituted 208, 76, 53 and 24, in addition to 217, 141, 215 and 14 individuals, respectively.

© 2023 Erickson P et al. *JAMA Neurology.*

eTable12. Cross-sectional tau PET analysis in ADNI.

	**Estimated mean**	**Standard error**	**P-value vs A-T-**	**P-value vs. A+T-**	**P-value vs. A+T+**	**P-value vs. A-T+**
A-T-	1.19	0.029	–	0.031	<0.001	0.408
A+T-	1.31	0.048	0.031	–	0.003	0.352
A+T+	1.54	0.038	<0.001	0.003	–	<0.001
A-T+	1.24	0.059	0.408	0.352	<0.001	–

The A-T-, A+T-, A+T+ and A-T+ profiles constituted 95, 29, 47 and 21 individuals, respectively. Linear models, including age, sex, APOEε4 status and years of education as covariates were used in both cohorts. A+/- indicates CSF Aβ42/40 binary status, and T+/- indicates CSF p-tau181 binary status.

© 2023 Erickson P et al. *JAMA Neurology.*

eTable13. Cross-sectional tau PET analysis in WISC

	**Estimated mean**	**Standard error**	**P-value vs. A-T-**	**P-value vs. A+T-**	**P-value vs. A+T+**	**P-value vs. A-T+**
A-T-	1.06	0.021	–	0.158	<.001	0.99
A+T-	1.42	0.057	0.16	–	0.091	0.132
A+T+	1.63	0.072	0.002	0.091	–	0.002
A-T+	1.13	0.117	0.99	0.132	0.002	–

The A-T-, A+T-, A+T+ and A-T+ profiles constituted 179, 26, 16 and 6 individuals, respectively. Linear models, including age, sex, APOEε4 status and years of education as covariates were used in both cohorts. A+/- indicates CSF Aβ42/40 binary status, and T+/- indicates CSF p-tau181 binary status.

© 2023 Erickson P et al. *JAMA Neurology.*

eFigure 1. Age-stratified prevalence of CSF AT profiles using CSF Aβ42 alone for "A".

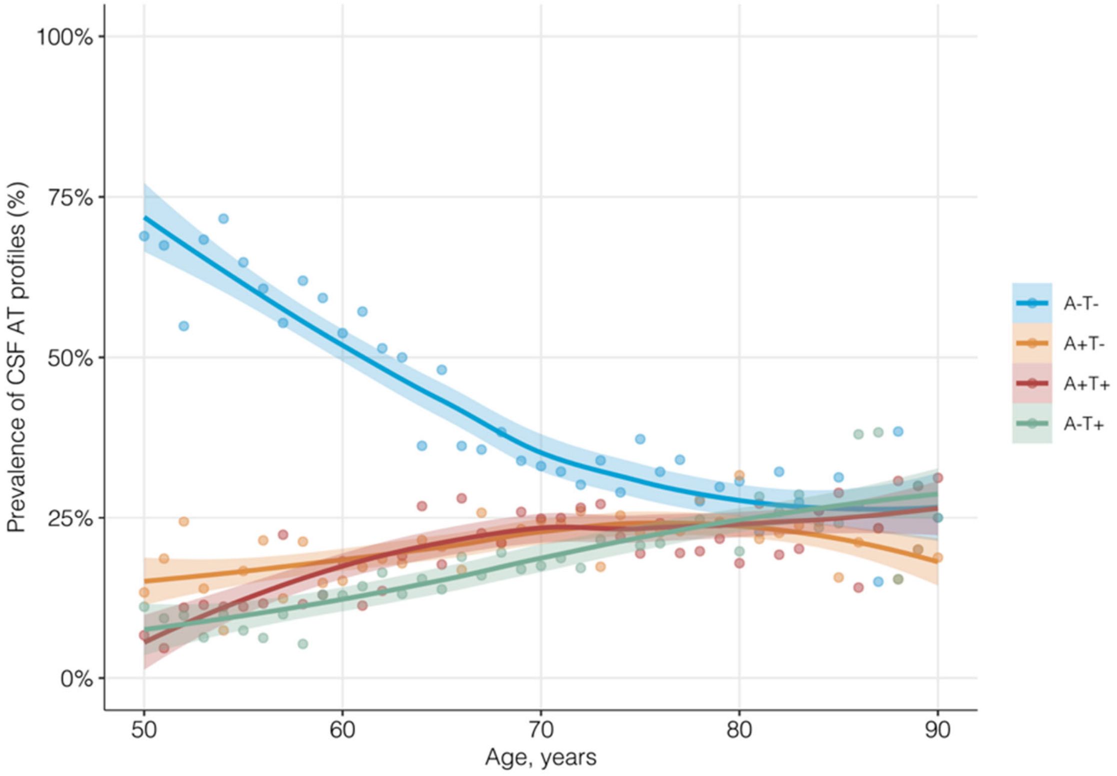

The colored dots represent the prevalence in % at each age (in years) of each biomarker category (blue, A-T-; orange, A+T-; red, A+T+; green, A-T+) based on the cut-offs used in clinical routine. The solid lines represent corresponding locally estimated scatterplot smoothing (LOESS) regression lines, with shaded areas indicating 95% CIs. This indicated that when using CSF Aβ42 alone, instead of the recommended CSF Aβ42/40 ratio, all three groups with abnormal biomarkers (A+T-, A+T+, and A-T+) present similar, nearly indistinct, trends for prevalence increases with increases in age.

© 2023 Erickson P et al. *JAMA Neurology.*

eFigure 2. Drift analyses in the UGOT dataset.

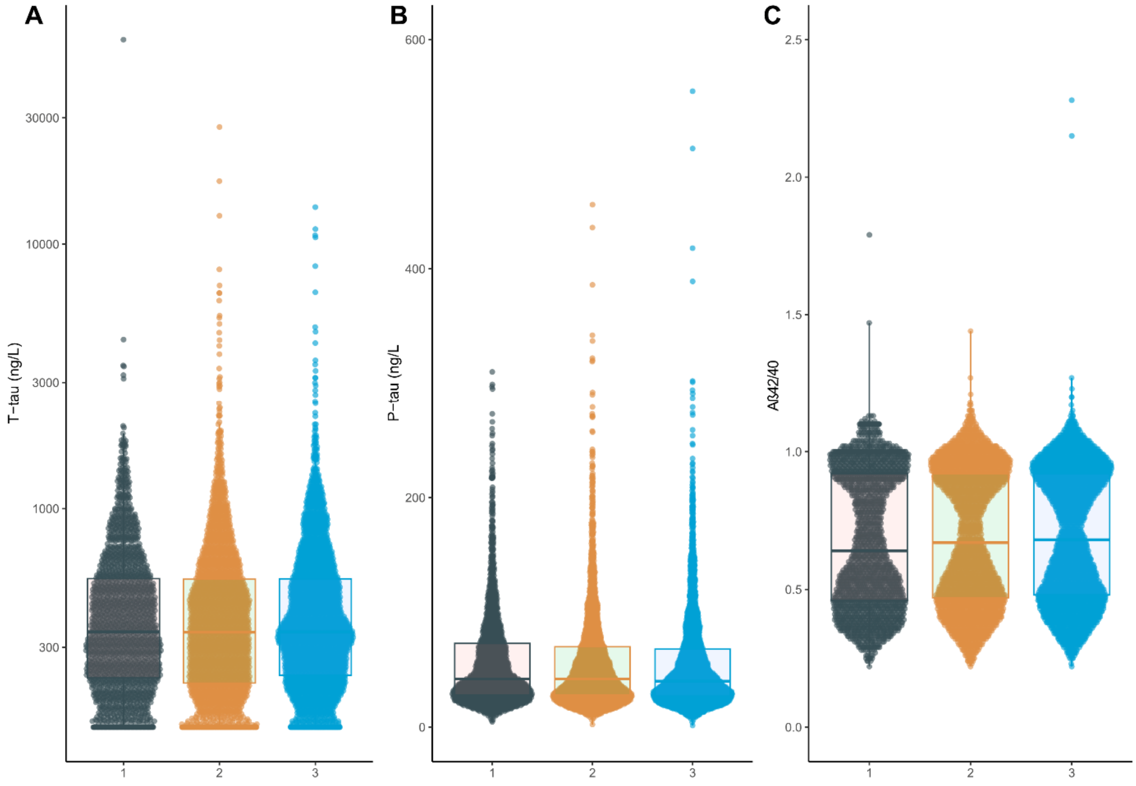

The graph displays group comparisons of (A) t-tau, (B) p-tau and (C) Aβ42/40*10 across time periods 1 (November 7th, 2019 – July 1st, 2020), 2 (July 2nd, 2020 – February 23rd, 2021), and 3 (February 24th, 2021 – January 18th, 2021) in the UGOT cohort. Group comparisons were performed using a one-way analysis of variance. A two-sided p-value <0.05 was considered significant. In panel A, concentrations of t-tau are presented on a $\log_{10}$ scale for visualization purposes. Also, for visualization purposes, three outliers with Aβ42/40*10 ratio > 2.6 was excluded from the graph but included in all statistical analyses.

© 2023 Erickson P et al. *JAMA Neurology.*

eReferences

1. Hansson O, Batrla R, Brix B, et al. The Alzheimer's Association international guidelines for handling of cerebrospinal fluid for routine clinical measurements of amyloid beta and tau. *Alzheimers Dement*. Sep 2021;17(9):1575-1582. doi:10.1002/alz.12316

2. Gobom J, Parnetti L, Rosa-Neto P, et al. Validation of the LUMIPULSE automated immunoassay for the measurement of core AD biomarkers in cerebrospinal fluid. *Clin Chem Lab Med*. Jan 27 2022;60(2):207-219. doi:10.1515/cclm-2021-0651

3. Korecka M, Figurski MJ, Landau SM, et al. Analytical and Clinical Performance of Amyloid-Beta Peptides Measurements in CSF of ADNIGO/2 Participants by an LC-MS/MS Reference Method. *Clin Chem*. Apr 1 2020;66(4):587-597. doi:10.1093/clinchem/hvaa012

4. Blennow K, Shaw LM, Stomrud E, et al. Predicting clinical decline and conversion to Alzheimer's disease or dementia using novel Elecsys Abeta(1-42), pTau and tTau CSF immunoassays. *Sci Rep*. Dec 13 2019;9(1):19024. doi:10.1038/s41598-019-54204-z

5. Van Hulle C, Jonaitis EM, Betthauser TJ, et al. An examination of a novel multipanel of CSF biomarkers in the Alzheimer's disease clinical and pathological continuum. *Alzheimers Dement*. Mar 2021;17(3):431-445. doi:10.1002/alz.12204

6. Jack CR, Jr., Wiste HJ, Weigand SD, et al. Defining imaging biomarker cut points for brain aging and Alzheimer's disease. *Alzheimers Dement*. Mar 2017;13(3):205-216. doi:10.1016/j.jalz.2016.08.005

7. Jagust WJ, Landau SM, Koeppe RA, et al. The Alzheimer's Disease Neuroimaging Initiative 2 PET Core: 2015. *Alzheimers Dement*. Jul 2015;11(7):757-71. doi:10.1016/j.jalz.2015.05.001

8. Wyman BT, Harvey DJ, Crawford K, et al. Standardization of analysis sets for reporting results from ADNI MRI data. *Alzheimers Dement*. May 2013;9(3):332-7. doi:10.1016/j.jalz.2012.06.004

9. Donohue MC, Sperling RA, Petersen R, et al. Association Between Elevated Brain Amyloid and Subsequent Cognitive Decline Among Cognitively Normal Persons. *JAMA*. Jun 13 2017;317(22):2305-2316. doi:10.1001/jama.2017.6669

10. Donohue MC, Sperling RA, Salmon DP, et al. The preclinical Alzheimer cognitive composite: measuring amyloid-related decline. *JAMA Neurol*. Aug 2014;71(8):961-70. doi:10.1001/jamaneurol.2014.803

11. Jonaitis EM, Koscik RL, Clark LR, et al. Measuring longitudinal cognition: Individual tests versus composites. *Alzheimers Dement (Amst)*. Dec 2019;11:74-84. doi:10.1016/j.dadm.2018.11.006

© 2023 Erickson P et al. *JAMA Neurology.*
