# Supplementary material for: Prevalence and Clinical Implications of a β-Amyloid–Negative, Tau-Positive Cerebrospinal Fluid Biomarker Profile in Alzheimer Disease
Source: JAMA Neurol. 2023 Jul 31;80(9):969–79. doi: 10.1001/jamaneurol.2023.2338 (PMC10391361; doi:10.1001/jamaneurol.2023.2338)
Supplement: Supplement 2. — Nonauthor Collaborators. ADNI Cohort. [file jamaneurol-e232338-s002.pdf]

Supplemental Online Content: Nonauthor Collaborators
*First name, last name, and suffix (if applicable) are required and will appear in PubMed.

*Group Name(s): ADNI Cohort							
***First Name and Middle Initial(s)**	***Last Name**	***Suffix (eg, Jr, III)**	Academic Degrees	Institution	Location (city, state/province, country)	Role or Contribution, eg, chair, principal investigator	Group (if more than 1 Group listed in the byline) and/or Subgroup (eg, Steering Committee)
Michael	Weiner						
Paul	Aisen						
Ronald	Petersen						
Clifford R.	Jack	Jr.					
William	Jagust						
John Q	Trojanowki						
Arthur W.	Toga						
Laurel	Beckett						
Robert C.	Green						
Andrew J.	Saykin						
John	Morris						
Leslie M.	Shaw						
Enchi	Liu						
Tom	Montine						
Ronald G.	Thomas						
Michael	Donohue						
Sarah	Walter						
Devon	Gessert						
Tamie	Sather						
Gus	Jiminez						
Danielle	Harvey						
Michael	Donohue						
Matthew	Bernstein						
Nick	Fox						
Paul	Thompson						
Norbert	Schuff						
Charles	DeCArli						
Bret	Borowski						
Jeff	Gunter						

Supplemental Online Content: Nonauthor Collaborators
*First name, last name, and suffix (if applicable) are required and will appear in PubMed.

***First Name and Middle Initial(s)**	***Last Name**	***Suffix (eg, Jr, III)**	Academic Degrees	Institution	Location (city, state/province, country)	Role or Contribution, eg, chair, principal investigator	Group (if more than 1 Group listed in the byline) and/or Subgroup (eg, Steering Committee)
Matt	Senjem						
Prashanthi	Vemuri						
David	Jones						
Kejal	Kantarci						
Chad	Ward						
Robert A.	Koeppe						
Norm	Foster						
Eric M.	Reiman						
Kewei	Chen						
Chet	Mathis						
Susan	Landau						
Nigel J.	Cairns						
Erin	Householder						
Lisa	Taylor Reinwald						
Virginia	Lee						
Magdalena	Korecka						
Michal	Figurski						
Karen	Crawford						
Scott	Neu						
Tatiana M.	Foroud						
Steven	Potkin						
Li	Shen						
Faber	Kelley						
Sungeun	Kim						
Kwangsik	Nho						
Zaven	Kachaturian						
Richard	Frank						
Peter J.	J Snyder						
Susan	Molchan						
Jeffrey	Kaye						

Supplemental Online Content: Nonauthor Collaborators
*First name, last name, and suffix (if applicable) are required and will appear in PubMed.

***First Name and Middle Initial(s)**	***Last Name**	***Suffix (eg, Jr, III)**	Academic Degrees	Institution	Location (city, state/province, country)	Role or Contribution, eg, chair, principal investigator	Group (if more than 1 Group listed in the byline) and/or Subgroup (eg, Steering Committee)
Joseph	Quinn						
Betty	Lind						
Raina	Carter						
Sara	Dolen						
Lon S.	Schneider						
Sonia	Pawluczyk						
Mauricio	Beccera						
Liberty	Teodoro						
Bryan M.	M Spann						
James	Brewer						
Helen	Vanderswag						
Adam	Fleisher						
Judith L	Heidebrink						
Joanne L	Lord						
Ronald	Petersen						
Sara	Mason						
Colleen	Albers						
David	Knopman						
Kris	Johnson						
Rachelle S	Doody						
Javier	Villanueva Meyer						
Munir	Chowdhury						
Susan	Rountree						
Mimi	Dang						
Yaakov	Stern						
Lawrence S	Honig						
Karen L	Bell						
Beau	Ances						
John C	Morris						
Maria	Carroll						

Supplemental Online Content: Nonauthor Collaborators
*First name, last name, and suffix (if applicable) are required and will appear in PubMed.

***First Name and Middle Initial(s)**	***Last Name**	***Suffix (eg, Jr, III)**	Academic Degrees	Institution	Location (city, state/province, country)	Role or Contribution, eg, chair, principal investigator	Group (if more than 1 Group listed in the byline) and/or Subgroup (eg, Steering Committee)
Sue	Leon						
Erin	Householder						
Mark A	Mintun						
Stacy	Schneider						
Angela	OliverNG						
Randall	Griffith						
David	Clark						
David	Geldmacher						
John	Brockington						
Erik	Roberson						
Hillel	Grossman						
Effie	Mitsis						
Leyla	deToledo-Morrell						
Raj C	Shah						
Ranjan	Duara						
Daniel	Varon						
Maria T	Greig						
Peggy	Roberts						
Marilyn	Albert						
Chiadi	Onyike						
Daniel	D’Agostino	II					
Stephanie	Kielb						
James E	Galvin						
Dana M	Pogorelec						
Brittany	Cerbone						
Christina A	Michel						
Henry	Rusinek						
Mony J	de Leon						
Lidia	Glodzik						
Susan	De Santi						

Supplemental Online Content: Nonauthor Collaborators
*First name, last name, and suffix (if applicable) are required and will appear in PubMed.

***First Name and Middle Initial(s)**	***Last Name**	***Suffix (eg, Jr, III)**	Academic Degrees	Institution	Location (city, state/province, country)	Role or Contribution, eg, chair, principal investigator	Group (if more than 1 Group listed in the byline) and/or Subgroup (eg, Steering Committee)
P	Murali Doraiswamy						
Jeffrey	R Petrella						
Terence	Z Wong						
Steven	E Arnold						
Jason	H Karlawish						
David	Wolk						
Charles	D Smith						
Greg	Jicha						
Peter	Hardy						
Partha	Sinha						
Elizabeth	Oates						
Gary	Conrad						
Oscar	Lopez						
MaryAnn	Oakley						
Donna	Simpson						
Anton	Porsteinsson						
Bonnie	Goldstein						
Kim	Martin						
Kelly	Makino						
M	Saleem Ismail						
Connie	Brand						
Ruth	Mulnard						
Gaby	Thai						
Catherine	Mc Adams Ortiz						
Kyle	Womack						
Dana	Mathews						
Mary	Quiceno						
Ramon	Diaz Arrastia						
Richard	King						

Supplemental Online Content: Nonauthor Collaborators
*First name, last name, and suffix (if applicable) are required and will appear in PubMed.

***First Name and Middle Initial(s)**	***Last Name**	***Suffix (eg, Jr, III)**	Academic Degrees	Institution	Location (city, state/province, country)	Role or Contribution, eg, chair, principal investigator	Group (if more than 1 Group listed in the byline) and/or Subgroup (eg, Steering Committee)
Myron	Weiner						
Kristen	Martin Cook						
Michael	DeVous						
Allan	Levey						
James	Lah						
Janet	Cellar						
Jeffrey	Burns						
Heather	Anderson						
Russell	Swerdlow						
Liana	Apostolova						
Kathleen	Tingus						
Ellen	Woo						
Daniel	Silverman						
Po	Lu						
George	Bartzokis						
Neill	Graff Radford						
Francine	ParfittH						
Tracy	Kendall						
Heather	Johnson						
Martin	Farlow						
Ann	Marie Hake						
Brandy	Matthews						
Scott	Herring						
Cynthia	Hunt						
Christopher	van Dyck						
Richard	Carson						
Martha G	MacAvoy						
Howard	Chertkow						
Howard	Bergman						
Chris	Hosein						
Sandra	Black						

Supplemental Online Content: Nonauthor Collaborators
*First name, last name, and suffix (if applicable) are required and will appear in PubMed.

***First Name and Middle Initial(s)**	***Last Name**	***Suffix (eg, Jr, III)**	Academic Degrees	Institution	Location (city, state/province, country)	Role or Contribution, eg, chair, principal investigator	Group (if more than 1 Group listed in the byline) and/or Subgroup (eg, Steering Committee)
Bojana	Stefanovic						
Curtis	Caldwell						
Ging	Yuek Robin Hsiung						
Howard	Feldman						
Benita	Mudge						
Michele	Assaly Past						
Andrew	Kertesz						
John	Rogers						
Dick	Trost						
Charles	Bernick						
Donna	Munic						
Diana	Kerwin						
Marek	Marsel Mesulam						
Kristine	Lipowski						
Chuang	Kuo Wu						
Nancy	Johnson						
Carl	Sadowsky						
Walter	Martinez						
Teresa	Villena						
Raymond	Scott Turner						
Kathleen	Johnson						
Brigid	Reynolds						
Reisa A	Sperling						
Keith A	Johnson						
Gad	Marshall						
Meghan	Frey						
Jerome	Yesavage						
Joy L	Taylor						
Barton	Lane						

Supplemental Online Content: Nonauthor Collaborators
*First name, last name, and suffix (if applicable) are required and will appear in PubMed.

***First Name and Middle Initial(s)**	***Last Name**	***Suffix (eg, Jr, III)**	Academic Degrees	Institution	Location (city, state/province, country)	Role or Contribution, eg, chair, principal investigator	Group (if more than 1 Group listed in the byline) and/or Subgroup (eg, Steering Committee)
Allyson	Rosen						
Jared	Tinklenberg						
Marwan N	Sabbagh						
Christine M	Belden						
Sandra A	Jacobson						
Sherye A	Sirrel						
Neil	Kowall						
Ronald	Killiany						
Andrew E	Budson						
Alexander	Norbash						
Patricia	Lynn Johnson						
Thomas O	Obisesan						
Saba	Wolday						
Joanne	Allard						
Alan	Lerner						
Paula	Ogrocki						
Leon	Hudson						
Evan	Fletcher						
Owen	Carmichael						
John	Olichney						
Charles	DeCarli						
Smita	Kittur						
Michael	Borrie						
T Y	Lee						
Rob	Bartha						
Sterling	Johnson						
Sanjay	Asthana						
Cynthia M	Carlsson						
Steven G	G Potkin						
Adrian	Preda						
Dana	Nguyen						

Supplemental Online Content: Nonauthor Collaborators
*First name, last name, and suffix (if applicable) are required and will appear in PubMed.

***First Name and Middle Initial(s)**	***Last Name**	***Suffix (eg, Jr, III)**	Academic Degrees	Institution	Location (city, state/province, country)	Role or Contribution, eg, chair, principal investigator	Group (if more than 1 Group listed in the byline) and/or Subgroup (eg, Steering Committee)
Pierre	Tariot						
Adam	Fleisher						
Stephanie	Reeder						
Vernice	Bates						
Horacio	Capote						
Michelle	Rainka						
Douglas W	Scharre						
Maria	Kataki						
Anahita	Adeli						
Earl A	Zimmerman						
Dzintra	Celmins						
Alice D	Brown						
Godfrey D	Pearlson						
Karen	Blank						
Karen	Anderson						
Robert B	Santulli						
Tamar J	Kitzmiller						
Eben S	Schwartz						
Kaycee M	SinkS						
Jeff D	Williamson						
Pradeep	Garg						
Franklin	Watkins						
Brian R	Ott						
Henry	Querfurth						
Geoffrey	Tremont						
Stephen	Salloway						
Paul	Malloy						
Stephen	Correia						
Howard J	Rosen						
Bruce L	Miller						
Jacobo	Mintzer						

Supplemental Online Content: Nonauthor Collaborators

*First name, last name, and suffix (if applicable) are required and will appear in PubMed.

***First Name and Middle Initial(s)**	***Last Name**	***Suffix (eg, Jr, III)**	Academic Degrees	Institution	Location (city, state/province, country)	Role or Contribution, eg, chair, principal investigator	Group (if more than 1 Group listed in the byline) and/or Subgroup (eg, Steering Committee)
Kenneth	Spicer						
David	Bachman						
Elizabether	Finger						
Stephen	Pasternak						
Irina	Rachinsky						
John	Rogers						
Andrew	Kertesz						
Dick	Drost						
Nunzio	Pomara						
Raymundo	Hernando						
Antero	Sarrael						
Susan K	Schultz						
Laura L	Boles Ponto						
Hyungsub	Shim						
Karen	Elizabeth Smith						
Norman	Relkin						
Gloria	Chaing						
Lisa	Raudin						
Amanda	Smith						
Kristin	Fargher						
Balebail	Ashok Raj						
