# Supplementary material for: Prevalence and Clinical Implications of a β-Amyloid–Negative, Tau-Positive Cerebrospinal Fluid Biomarker Profile in Alzheimer Disease
Source: JAMA Neurol. 2023 Jul 31;80(9):969–79. doi: 10.1001/jamaneurol.2023.2338 (PMC10391361; doi:10.1001/jamaneurol.2023.2338)
Supplement: Supplement 3. — Data Sharing Statement [file jamaneurol-e232338-s003.pdf]

Data Sharing Statement

Erickson. Prevalence and Clinical Implications of a β-Amyloid–Negative, Tau-Positive Cerebrospinal Fluid Biomarker Profile in Alzheimer Disease. *JAMA Neurol*. Published July 31, 2023. doi:10.1001/jamaneurol.2023.2338

Data
Data available: No

Additional Information
Explanation for why data not available: Deidentified data from the Wisconsin cohort are available by request through its website. All requests will be reviewed by study leadership, and if approved, data will be transferred subject to an institutional data use agreement. The UGOT data are available upon reasonable request made to the senior author and should agree with EU legislation on the general data protection regulation and decisions by the Ethical Review Board of Sweden and should be regulated in a material transfer agreement.
